# Classical and Bayesian Inference of an Exponentiated Half-Logistic Distribution under Adaptive Type II Progressive Censoring

**DOI:** 10.3390/e23121558

**Published:** 2021-11-23

**Authors:** Ziyu Xiong, Wenhao Gui

**Affiliations:** Department of Mathematics, Beijing Jiaotong University, Beijing 100044, China; 19271109@bjtu.edu.cn

**Keywords:** adaptive type-II progressive censoring, exponentiated half-logistic distribution, maximum likelihood estimation, Bayesian estimation, importance sampling, Lindley method, bootstrap method, Monte Carlo simulation

## Abstract

The point and interval estimations for the unknown parameters of an exponentiated half-logistic distribution based on adaptive type II progressive censoring are obtained in this article. At the beginning, the maximum likelihood estimators are derived. Afterward, the observed and expected Fisher’s information matrix are obtained to construct the asymptotic confidence intervals. Meanwhile, the percentile bootstrap method and the bootstrap-t method are put forward for the establishment of confidence intervals. With respect to Bayesian estimation, the Lindley method is used under three different loss functions. The importance sampling method is also applied to calculate Bayesian estimates and construct corresponding highest posterior density (HPD) credible intervals. Finally, numerous simulation studies are conducted on the basis of Markov Chain Monte Carlo (MCMC) samples to contrast the performance of the estimations, and an authentic data set is analyzed for exemplifying intention.

## 1. Introduction

### 1.1. Adaptive Type II Progressive Censoring Scheme

In this day and age, owing to the development of science and technology, industrial products have become greatly reliable and as a result, getting sufficient failure time during a life testing experiment for any statistical analysis purposes results in a sharp increase in cost and time. Hence, the aim of reducing test time and saving the cost leads us into the realm of censoring. With units removed before their failure time purposefully, the duration and cost can be greatly reduced. Many statisticians have investigated various censoring schemes. The two most commonly used censoring schemes are type I and type II censoring schemes. In type I censoring, the life-testing experiment terminates at a predetermined time while, under type II censoring, the life-testing test stops once the observed failure units reach the predetermined number. For the sake of further reducing the experimental cost and time, a concoction of these two schemes called hybrid censoring was put forward. However, none of these schemes permits the survival units to be removed during the experiment, which lacks flexibility. Accordingly, the concept of progressive censoring was brought forward by [1] to increase the flexibility of removing units other than the terminal experimental time. A concise presentation of the progressive type II censoring is as follows. Assume that there are totally *n* identical units in the test. In addition, the failure time of the units is defined as $X=(X_{\left(1:m:n\right)},X_{\left(2:m:n\right)},\cdots ,X_{\left(m-1:m:n\right)},X_{\left(m:m:n\right)})$, and the censoring scheme denotes as $R=(R_{1},R_{2},\cdots ,R_{m-1},R_{m})$, where $n-m=\sum_{i=1}^{m}R_{i}$. When the first unit fails at $X_{1}$, we remove $R_{1}$ units from n−1 units remained randomly. Then, we remove $R_{j}$ units from the $n-j-\sum_{i=1}^{j-1}R_{i}$ remaining units on the occurrence of the *j*-th failure in the same way. In addition, you can refer to [1,2] for further information in progressive censoring.

**Figure 1:**
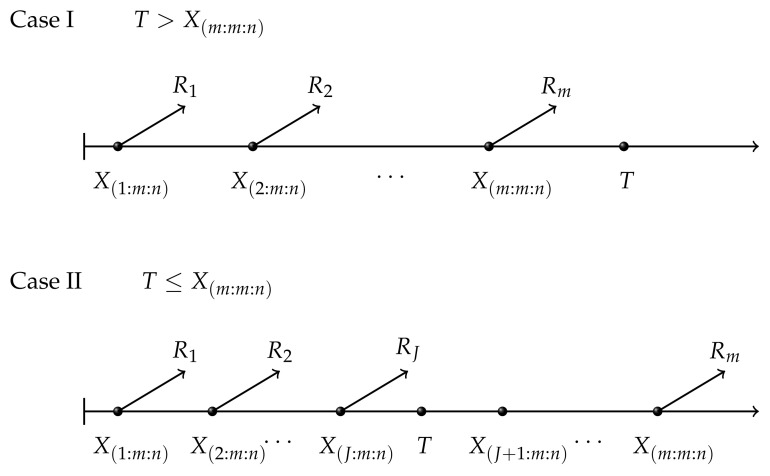
Adaptive type II progressive censoring.

However, one of the drawbacks of the scheme is that researchers can not control the experiment time in practical terms. Recently, Ref. [3] proposed a new censoring called adaptive type II progressive censoring scheme in the interest of saving the aggregate time and improving the analysis efficiency. Based on progressive type II censoring, the expected total experimental time *T* is also pre-fixed before the test. If $T>X_{\left(m:m:n\right)}$, the experiment is implemented according to the progressive type II censoring scheme with $R=(R_{1},R_{2},\cdots ,R_{m-1},R_{m})$ and terminates at time $X_{\left(m:m:n\right)}$. However, once the actual time runs over *T*, namely $T<X_{\left(m:m:n\right)}$, we do not stop the test at *T* but no longer remove survival units after the prefixed time *T*. Suppose that the time runs over *T* right after the occurrence of the *J*-th failure, namely $J=\max\{j,T>X_{\left(j:m:n\right)}\}$. Therefore, once the concrete test time runs over *T*, the censoring scheme after time *T* becomes $R_{J+1}=R_{J+2}=\cdots =R_{m-1}=0,\;R_{m}=n-m-\sum_{i=1}^{J}R_{i}$. In particular, there are two special situations with the change of *T*. If T=∞, the scheme eventually turns into progressive type II censoring. In addition, if the expected time *T* equals to 0, the scheme changes into the common type II censoring scheme. Figure 1 presents adaptive type II progressive censoring.

Since the adaptive type II progressive censoring scheme was proposed, its good property has attracted a great number of researchers to study this field. The adaptive progressive type II censoring model was further studied in Ref. [4]. Under this censoring model, Ref. [5] also studied the estimator of unknown parameters of Weibull distribution. The classical estimations and the Bayesian estimations were both derived from the scheme. The adaptive type II progressive censoring was collaborated with the exponential step-stress accelerated life-testing model to derive confidence intervals in Ref. [6]. Furthermore, this censoring scheme was also extended by taking account of competing risks under two-Parameter Rayleigh Distribution and making classical and Bayesian inference by Ref. [7].

### 1.2. The Exponentiated Half-Logistic Distribution

The exponentiated half-logistic distribution (EHL) is extremely famous in numerous applications particularly in parameter estimates. It has been applied in many areas, including insurance, engineering, medicine, education, etc. This distribution is suitable for modeling lifetime data and is extremely parallel to the two-parameter family of distributions, which is noted in Ref. [8]. For example, the Gamma distribution is an important distribution in the two-parameter family of distributions. However, compared to the Gamma distribution, exponentiated half-logistic distribution has a whip hand due to the closed form of its cumulative distribution.

In this article, we focus on the exponentiated half-logistic distribution. The probability density function (PDF) is written as:

(1) $$f\left(x;\lambda ,\sigma \right)=\frac{\lambda }{\sigma }{\left(\frac{1-e^{-\frac{x}{\sigma }}}{1+e^{-\frac{x}{\sigma }}}\right)}^{\lambda -1}\frac{2e^{-\frac{x}{\sigma }}}{{\left(1+e^{\frac{-x}{\sigma }}\right)}^{2}},\;\;x>0,\;\lambda ,\sigma >0,$$

and the cumulative distribution function (CDF) is described as

(2) $$F\left(x;\lambda ,\sigma \right)={\left(\frac{1-e^{-\frac{x}{\sigma }}}{1+e^{-\frac{x}{\sigma }}}\right)}^{\lambda },\;\;x>0,\;\lambda ,\sigma >0,$$

where λ>0 is the shape parameter and σ>0 is the scale parameter. We denote this distribution as EHI(λ,σ).

The corresponding reliability function is written as:

(3) $$R\left(t\right)=1-{\left(\frac{1-e^{-t\sigma }}{1+e^{-t\sigma }}\right)}^{\lambda },\;\;t>0,$$

while the hazard rate function is:

(4) $$h\left(t\right)=\frac{2\sigma \lambda e^{-t\sigma }}{{\left(1+e^{-t\sigma }\right)}^{2}\left[1-{\left(\frac{1-e^{-t\sigma }}{1+e^{-t\sigma }}\right)}^{\lambda }\right]}{\left(\frac{1-e^{-t\sigma }}{1+e^{-t\sigma }}\right)}^{\lambda -1},\;\;t>0.$$

**Figure 2:**
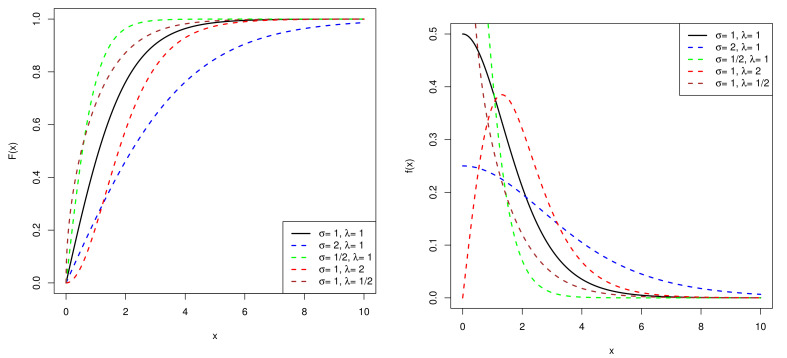
CDF (**left**) and PDF (**right**) of exponentiated half-logistic distribution.

From Figure 2, when $\frac{\lambda }{\sigma }>1$, the PDF of the exponentiated half-logistic distribution is unimodal. In addition, while $\frac{\lambda }{\sigma }<1$, it becomes monotonically decreasing. When λ is fixed, the smaller σ is, the more sharply the PDF decreases. As for the CDF of the distribution, the growth of CDF becomes slow with σ increasing. Furthermore, smaller λ results in a higher rising rate.

When λ=1, the exponentiated half-logistic distribution degrades into the renowned half logistic distribution. The half logistic distribution has extensive use particularly employed in censored data in the area of survival analysis. This distribution has been studied by some researchers. The order statistics of the half logistic distribution was studied in Ref. [9]. On the basis of progressively type II censored data, Ref. [10] derived the classical and Bayes estimators of the scale parameter of this distribution. In accordance with the study results of [10], analytic expressions were studied further for the biases of the maximum likelihood estimators of the distribution in [11]. The generalized ranked-set sampling technique was employed for obtaining parameters estimation of the half-logistic distribution in [12].

The exponentiated half-logistic distribution has recently attracted a lot of researchers. On the basis of progressive Type-II censored data, Ref. [13] derived the maximum likelihood estimator of the scale parameter in an exponentiated half logistic distribution and proposed some approximate maximum likelihood estimators as well. In addition to the MLE, Ref. [14] focused on the moment estimators and entropy estimator in this distribution. For the purpose of promoting practicability of the distribution, Ref. [15] extended the exponentiated half-logistic distribution by putting forward the concept of the exponentiated half-logistic family, which is a fresh generator of continuous distributions of two excess parameters. Considering that the life test sometimes stops at a pre-determined time, Ref. [16] developed acceptance sampling for the percentiles of this distribution. Meanwhile, not only the operating characteristic values of the sampling plans but also the producer’s risk were shown. Based on the distribution, Ref. [17] proposed an attribute control chart for time truncated life tests with different shape parameters. Thus far, research associated with this distribution has a great deal of space to explore.

In this article, the problem of the point and interval estimation of the parameters for exponentiated half logistic distribution under adaptive type II progressive censored data are considered. We organize the remainder paper as follows. In Section 2, the maximum likelihood estimates are derived and computed. Meanwhile, the observed and expected Fisher information matrix is acquired and then the asymptotic confidence intervals are established. We employ the bootstrap resampling method to build two bootstrap confidence intervals in Section 3. As for Section 4, Bayesian estimations under several loss functions are carried out by utilizing the Lindley method. The importance sampling method is also used to calculate the Bayesian estimates and construct the highest posterior density (HPD) credible intervals. Simulations are conducted and the behaviors of estimators obtained with the diverse methods are evaluated and compared in Section 5. An authentic data set is studied to illustrate the effectiveness of estimation means in the above sections in Section 6. In the end, the conclusions of point and interval estimations are drawn in Section 7.

## 2. Maximum Likelihood Estimation

### 2.1. Point Estimation

In this section, maximum likelihood estimation is used to estimate the unknown parameters on the basis of the adaptive type II progressive censored data. Assume that the adaptive type II progressive censored data come from an exponentiated half-logistic distribution. Let $x_{\left(i:m:n\right)}$ denote the *i*-th observation, thus we know $x_{\left(1:m:n\right)}<x_{\left(2:m:n\right)}\ldots <x_{\left(m:m:n\right)}$. In addition, *T* represents the expected experimental time and *J* denotes the index of the last failure before time *T*.

For the sake of simplicity, let $\underline{x}=\left(x_{1},x_{2},\cdots ,x_{m}\right)$ denote $\left(x_{\left(1:m:n\right)},x_{\left(2:m:n\right)},\cdots ,x_{\left(m:m:n\right)}\right)$. The likelihood function turns to be

(5) $$L\left(\lambda ,\sigma |\underline{x}\right)=D_{J}{\left[1-F\left(x_{m}\right)\right]}^{n-m-\sum_{i=1}^{J}R_{i}}\prod_{i=1}^{J}{\left[1-F\left(x_{i}\right)\right]}^{R_{i}}\prod_{i=1}^{m}f\left(x_{i}\right),$$

where

$$D_{J}=\prod_{i=1}^{m}\left(n+1-i-\sum_{k=1}^{\min\{J,i-1\}}R_{k}\right).$$

The corresponding likelihood function is derived as

(6) $$\begin{array}{ccc} L(\lambda ,\sigma |\underline{x}) & = & D_{J}\sigma^{-m}\lambda^{m}e^{-\lambda \sum_{i=1}^{m}ln\frac{1+e^{-\frac{x_{i}}{\sigma }}}{1-e^{-\frac{x_{i}}{\sigma }}}-\frac{1}{\sigma }\sum_{i=1}^{m}x_{i}}\prod_{i=1}^{m}\frac{1}{1-e^{-\frac{2x_{i}}{\sigma }}}\\ & & \times \prod_{i=1}^{J}{\left[1-{\left(\frac{1-e^{-\frac{x_{i}}{\sigma }}}{1+e^{-\frac{x_{i}}{\sigma }}}\right)}^{\lambda }\right]}^{R_{i}}{\left[1-{\left(\frac{1-e^{-\frac{x_{m}}{\sigma }}}{1+e^{-\frac{x_{m}}{\sigma }}}\right)}^{\lambda }\right]}^{n-m-\sum_{i=1}^{J}R_{i}}. \end{array}$$

Therefore, the log-likelihood function can be obtained by

(7) $$\begin{array}{ccc} l(\lambda ,\sigma |\underline{x}) & = & D+m\ln\lambda -m\ln\sigma -\frac{\sum_{i=1}^{m}x_{i}}{\sigma }-\lambda \sum_{i=1}^{m}\ln\frac{1+e^{-\frac{x_{i}}{\sigma }}}{1-e^{-\frac{x_{i}}{\sigma }}}+\sum_{i=1}^{m}\ln\frac{1}{1-e^{\frac{-2x_{i}}{\sigma }}}\\ & & +\sum_{i=1}^{J}R_{i}\ln\left(1-F\left(x_{i}\right)\right)+\left(n-m-\sum_{i=1}^{J}R_{i}\right)\ln\left(1-F\left(x_{m}\right)\right), \end{array}$$

where *D* is a constant.

Finding the partial derivatives involving σ and λ separately and letting them equal zero, the equations correspond to   

(8) $$\frac{\partial l}{\partial \sigma }=-\frac{1}{\sigma }\left[m+\left(1-\frac{1}{\lambda }\right)\sum_{i=1}^{m}\zeta_{i}x_{i}-\frac{1}{\sigma }\sum_{i=1}^{m}{\left(F\left(x_{i}\right)\right)}^{\frac{1}{\lambda }}x_{i}-\sum_{i=1}^{J}R_{i}\eta_{i}x_{i}-\left(n-m-\sum_{i=1}^{J}\right)\eta_{m}x_{m}\right]=0,$$

(9) $$\frac{\partial l}{\partial \lambda }=\frac{1}{\lambda }\left[m+\sum_{i=1}^{m}\ln F\left(x_{i}\right)-\sum_{i=1}^{J}R_{i}G_{i}F\left(x_{i}\right)-\left(n-m-\sum_{i=1}^{J}R_{i}\right)G_{m}F\left(x_{m}\right)\right]=0,$$

where $\zeta_{i}=\frac{f(x_{i})}{F(x_{i})}$, $\eta_{i}=\frac{f(x_{i})}{1-F(x_{i})}$, $G_{i}=\frac{\ln F(x_{i})}{1-F(x_{i})}$.

The roots of the equations correspond to the MLEs. However, owing to the nonlinearity of the equations, obviously we can not obtain the explicit expressions. Thus, the Newton–Raphson method is employed to solve this problem. The Newton–Raphson method is an important method to find the roots of equations by employing the Taylor series method. Thus, the Newton–Raphson method is employed to acquire the MLEs, written as $\hat{\sigma }$ and $\hat{\lambda }$.

### 2.2. Asymptotic Confidence Interval

In this subsection, the asymptotic confidence intervals for σ and λ are established by employing $Var(\hat{\sigma })$ and $Var(\hat{\lambda })$. We acquire the asymptotic confidence intervals for σ and λ from the variance–covariance matrix, which is also known as the inverse Fisher information matrix. The Fisher information matrix is a generalization of the Fisher information amount. The Fisher information amount represents the average amount of information about the state parameters in a certain sense that a sample of random variables can provide. The Fisher information matrix (FIM) $I\left(\sigma ,\lambda \right)$ is

(10) $$I\left(\sigma ,\lambda \right)=-E\left[\begin{array}{cc} \frac{\partial^{2}l\left(\lambda ,\sigma \right)}{\partial \sigma^{2}} & \frac{\partial^{2}l\left(\lambda ,\sigma \right)}{\partial \lambda \partial \sigma }\\ \frac{\partial^{2}l\left(\lambda ,\sigma \right)}{\partial \lambda \partial \sigma } & \frac{\partial^{2}l\left(\lambda ,\sigma \right)}{\partial \lambda^{2}} \end{array}\right].$$

Here,

(11) $$\frac{\partial^{2}l}{\partial \lambda^{2}}=-\frac{1}{\lambda^{2}}\left[m+\sum_{i=1}^{J}R_{i}G_{i}^{2}F\left(x_{i}\right)+\left(n-\sum_{i=1}^{J}R_{i}-m\right)F\left(x_{m}\right)G_{m}^{2}\right],$$

(12) $$\frac{\partial^{2}l}{\partial \lambda \partial \sigma }=\frac{1}{\lambda \sigma }\left[-\sum_{i=1}^{m}\zeta_{i}x_{i}+\sum_{i=1}^{J}R_{i}x_{i}\eta_{i}\left(1+G_{i}\right)+\left(n-m-\sum_{i=1}^{J}R_{i}\right)x_{m}\eta_{m}\left(1+G_{m}\right)\right],$$

(13) $$\begin{array}{ccc} \frac{\partial^{2}l}{\partial \sigma^{2}} & = & -\frac{1}{\sigma^{2}}\{m+\left(1-\frac{1}{\lambda }\right)\sum_{i=1}^{m}x_{i}\left[\left(1-H_{i}\right)\zeta_{i}-\zeta^{2}\right]-\frac{1}{\sigma }\sum_{i=1}^{m}x_{i}F{\left(x_{i}\right)}^{\frac{1}{\lambda }}\left(2+\frac{1}{\lambda }\zeta_{i}\right)\\ & & +\sum_{i=1}^{J}\left[-\eta_{i}^{2}+\left(H_{i}-1\right)\eta_{i}\right]R_{i}+\left(n-m-\sum_{i=1}^{J}R_{i}\right)\left[-\eta_{m}^{2}+\left(H_{m}-1\right)\eta_{m}\right]x_{m}\}, \end{array}$$

where $H_{i}=-1+\frac{x_{i}}{\sigma }F{\left(x_{i}\right)}^{\frac{1}{\lambda }}+\left(-1+\lambda \right)\frac{x_{i}}{\lambda }\zeta_{i}$.

The FIM $I\left(\sigma ,\lambda \right)$ is called the expected Fisher matrix. It is determined by the distribution of the order statistics $X_{\left(i\right)}$. The PDF of $X_{\left(i\right)}$ based on the progressive type II censored sample generally can be derived from [1].

(14) $$f_{x_{\left(i\right)}}\left(x_{\left(i\right)}\right)=c_{i-1}^{0}\sum_{k=1}^{i}d_{k,i}^{0}f\left(x_{\left(i\right)}\right){\left[1-F\left(x_{i}\right)\right]}^{r_{k}^{0}-1},$$

where

$$\begin{array}{ccc} & & c_{i-1}^{0}=\prod_{k=1}^{i}r_{k}^{0},\;r_{i}^{0}=m+1-i+\sum_{k=i}^{m}R_{k},i=1,2,\cdots ,j,\\ & & d_{11}^{0}=1,\;d_{k,i}^{0}=\prod_{h=1,h\neq k}^{i}\frac{1}{r_{h}^{0}-r_{k}^{0}},1\leq k\leq i\leq j. \end{array}$$

The adaptive progressive type II censoring is considered as an improvement of the progressive type II censoring. Actually, the PDF of $X_{\left(i\right)}$ of EHL(λ,σ) under adaptive progressive type II censoring turns out to be

(15) $$f_{x_{\left(i\right)}}\left(x_{\left(i\right)}\right)=\frac{c_{i-1}^{1}}{c_{j-1}^{1}}\sum_{k=j+1}^{i}d_{k,i}^{1}v\left(x_{\left(i\right)}\right){\left[1-V\left(x_{\left(i\right)}\right)\right]}^{r_{k}^{1}-1},$$

where

$$\begin{array}{ccc} & & c_{i-1}^{1}=\prod_{k=1}^{i}r_{k}^{1},\;r_{i}^{1}=n-i+1-\sum_{k=1}^{j}R_{k},i=j+1,j+2,\cdots ,m,\;d_{j+1,j+1}^{1}=1,\\ & & d_{k,i}^{1}=\prod_{h=j+1,h\neq k}^{i}\frac{1}{r_{h}^{1}-r_{k}^{1}},j+1\leq k\leq i\leq m,\;v\left(x_{\left(i\right)}\right)=\frac{f(x_{\left(i\right)})}{1-F(x_{\left(j\right)})},\;V\left(x_{\left(i\right)}\right)=\frac{F\left(x_{\left(i\right)}\right)-F\left(x_{\left(j\right)}\right)}{1-F(x_{\left(j\right)})}. \end{array}$$

After sorting out, the formula (15) can be written as   

(16) $$f_{x_{\left(i\right)}}\left(x_{\left(i\right)}\right)=\left\{\begin{array}{c} c_{i-1}^{0}\sum_{k=1}^{i}d_{k,i}^{0}\frac{\lambda }{\sigma }{\left(\frac{1-e^{-\frac{x_{\left(i\right)}}{\sigma }}}{1+e^{-\frac{x_{\left(i\right)}}{\sigma }}}\right)}^{\lambda -1}\frac{2e^{-\frac{x_{\left(i\right)}}{\sigma }}}{{\left(1+e^{\frac{-x_{\left(i\right)}}{\sigma }}\right)}^{2}}{\left[1-{\left(\frac{1-e^{-\frac{x_{\left(i\right)}}{\sigma }}}{1+e^{-\frac{x_{\left(i\right)}}{\sigma }}}\right)}^{\lambda }\right]}^{r_{k}^{0}-1}\;,i=1,2,\cdots ,j,\\ \frac{c_{i-1}^{1}}{c_{j-1}^{1}}\sum_{k=j+1}^{i}d_{k,i}^{1}\frac{\frac{\lambda }{\sigma }{\left(\frac{1-e^{-\frac{x_{\left(i\right)}}{\sigma }}}{1+e^{-\frac{x_{\left(i\right)}}{\sigma }}}\right)}^{\lambda -1}\frac{2e^{-\frac{x_{\left(i\right)}}{\sigma }}}{{\left(1+e^{\frac{-x_{\left(i\right)}}{\sigma }}\right)}^{2}}}{1-{\left(\frac{1-e^{-\frac{x_{\left(i\right)}}{\sigma }}}{1+e^{-\frac{x_{\left(i\right)}}{\sigma }}}\right)}^{\lambda }}{\left[\frac{1-{\left(\frac{1-e^{-\frac{x_{\left(i\right)}}{\sigma }}}{1+e^{-\frac{x_{\left(i\right)}}{\sigma }}}\right)}^{\lambda }}{1-{\left(\frac{1-e^{-\frac{x_{\left(i\right)}}{\sigma }}}{1+e^{-\frac{x_{\left(i\right)}}{\sigma }}}\right)}^{\lambda }}\right]}^{r_{k}^{1}-1}\;,i=j+1,j+2,\cdots ,m. \end{array}\right.$$

Afterwards, we can calculate Fisher information matrix FIM I(σ,λ) directly based on (16). In order to simplify complex calculation, the observed Fisher Information matrix $I\left(\hat{\sigma },\hat{\lambda }\right)$ is employed skillfully to approximate the expected Fisher information matrix, and then the variance–covariance matrix can be obtained. Then, the $I(\hat{\sigma },\hat{\lambda })$ turns out to be

(17) $$I\left(\hat{\sigma },\hat{\lambda }\right)=-{\left[\begin{array}{cc} \frac{\partial^{2}l\left(\lambda ,\sigma \right)}{\partial \sigma^{2}} & \frac{\partial^{2}l\left(\lambda ,\sigma \right)}{\partial \lambda \partial \sigma }\\ \frac{\partial^{2}l\left(\lambda ,\sigma \right)}{\partial \lambda \partial \sigma } & \frac{\partial^{2}l\left(\lambda ,\sigma \right)}{\partial \lambda^{2}} \end{array}\right]}_{\left(\sigma ,\lambda \right)=\left(\hat{\sigma },\hat{\lambda }\right)}.$$

Here, $\hat{\sigma }$ and $\hat{\lambda }$ are the MLEs of σ and λ separately.

Then, the asymptotic variance–covariance matrix is the inverse of the observed Fisher Information matrix $I\left(\hat{\sigma },\hat{\lambda }\right)$, denoted as $I^{-1}\left(\hat{\sigma },\hat{\lambda }\right)$.

(18) $$I^{-1}\left(\hat{\sigma },\hat{\lambda }\right)=\left[\begin{array}{cc} Var(\hat{\sigma })\; & Cov(\hat{\sigma },\hat{\lambda })\\ Cov(\hat{\lambda },\hat{\sigma }) & Var(\hat{\lambda }) \end{array}\right].$$

Thus, the $100\left(1-\alpha \right)$% asymptotic confidence intervals for σ and λ can be constructed as

$$\left(\hat{\sigma }-d_{\frac{\alpha }{2}}\times \sqrt{Var\left(\hat{\sigma }\right)},\hat{\sigma }+d_{\frac{\alpha }{2}}\times \sqrt{Var\left(\hat{\sigma }\right)}\right)$$

and

$$\left(\hat{\lambda }-d_{\frac{\alpha }{2}}\times \sqrt{Var(\hat{\lambda })},\hat{\lambda }+d_{\frac{\alpha }{2}}\times \sqrt{Var(\hat{\lambda })}\right)$$

where $d_{\alpha }$ denotes the upper α-th quantile of the standard normal distribution.

## 3. Bootstrap Confidence Intervals

It is noticed that the classical theory works well with a large sample size while it makes little sense on the condition that the sample size is small. Thus, the bootstrap methods are applied to provide more precise confidence intervals.

The two most commonly used bootstrap methods are proposed, see [18]. One is the percentile bootstrap method (boot-p). It replaces the distribution of original sample statistics with the distribution of Bootstrap sample statistics to establish confidence intervals. The other is the bootstrap-t method (boot-t). In addition, the core idea of this method is to convert the Bootstrap sample statistic into the corresponding t statistic. The detailed procedure for simulation of the two bootstrap methods is listed, see Algorithms 1 and 2.
**Algorithm 1:** Constructing percentile bootstrap confidence intervalsstep 1Set the simulation number $N_{boot}$ times ahead.step 2Compute the MLEs of σ and λ under the original censored sample $\underline{x}=\left(x_{1},x_{2},\cdots ,x_{m}\right)$, denoted as $\hat{\sigma }$ and $\hat{\lambda }$. (If we carry out a simulation study, we should first generate an adaptive progressive type II censored sample $\underline{x}=\left(x_{1},x_{2},\cdots ,x_{m}\right)$ from EHL(λ,σ) with T,n,m,R as the original sample.)step 3Generate a bootstrap sample ${\underline{x}}^{*}$ using $\hat{\sigma },\hat{\lambda }$ and the same censoring pattern (n,m,T,R). Then, calculate the bootstrap MLEs under sample ${\underline{x}}^{*}$, denote as ${\hat{\sigma }}^{*}$ and ${\hat{\lambda }}^{*}$.step 4Repeat step 3 $N_{boot}$ times, then we can obtain a series of bootstrap MLEs$\left({\hat{\sigma }}_{**}^{\left(1\right)},{\hat{\sigma }}_{**}^{\left(2\right)},\cdots ,{\hat{\sigma }}_{**}^{\left(N_{boot}\right)}\right)$ and $\left({\hat{\lambda }}_{**}^{\left(1\right)},{\hat{\lambda }}_{**}^{\left(2\right)},\cdots ,{\hat{\lambda }}_{**}^{\left(N_{boot}\right)}\right)$.step 5Arrange $\left({\hat{\sigma }}_{**}^{\left(1\right)},{\hat{\sigma }}_{**}^{\left(2\right)},\cdots ,{\hat{\sigma }}_{**}^{\left(N_{boot}\right)}\right)$ and $\left({\hat{\lambda }}_{**}^{\left(1\right)},{\hat{\lambda }}_{**}^{\left(2\right)},\cdots ,{\hat{\lambda }}_{**}^{\left(N_{boot}\right)}\right)$ in ascending order, respectively, and obtain $\left({\hat{\sigma }}_{**}^{\left[1\right]},{\hat{\sigma }}_{**}^{\left[2\right]},\cdots ,{\hat{\sigma }}_{**}^{\left[N_{boot}\right]}\right)$ and $\left({\hat{\lambda }}_{**}^{\left[1\right]},{\hat{\lambda }}_{**}^{\left[2\right]},\cdots ,{\hat{\lambda }}_{**}^{\left[N_{boot}\right]}\right)$.

### 3.1. Percentile Bootstrap Confidence Intervals

Then, the $100\left(1-\alpha \right)\%$ Boot-p confidence intervals are given by $\left({\hat{\sigma }}_{**}^{\left[K_{1}\right]},{\hat{\sigma }}_{**}^{\left[K_{2}\right]}\right)$ and $\left({\hat{\lambda }}_{**}^{\left[K_{1}\right]},{\hat{\lambda }}_{**}^{\left[K_{2}\right]}\right)$, where $K_{1}$ and $K_{2}$ are the integer parts of $\frac{\alpha }{2}\times N_{boot}$ and $\left(1-\frac{\alpha }{2}\right)\times N_{boot}$, respectively.

### 3.2. Bootstrap-t Confidence Intervals

Then, the $100\left(1-\alpha \right)\%$ Boot-t confidence intervals are given by

$$\left(\hat{\sigma }-{\tilde{S_{1}}}_{**}^{\left[K_{2}\right]}\sqrt{Var(\hat{\sigma })},\hat{\sigma }-{\tilde{S_{1}}}_{**}^{\left[K_{1}\right]}\sqrt{Var(\hat{\sigma })}\right)$$

and

$$\left(\hat{\lambda }-{\tilde{S_{1}}}_{**}^{\left[K_{2}\right]}\sqrt{Var(\hat{\lambda })},\hat{\lambda }-{\tilde{S_{1}}}_{**}^{\left[K_{1}\right]}\sqrt{Var(\hat{\lambda })}\right)$$

where $K_{1}$ and $K_{2}$ are the integer parts of $\frac{\alpha }{2}\times N_{boot}$ and $\left(1-\frac{\alpha }{2}\right)\times N_{boot}$, respectively.
**Algorithm 2:** Constructing bootstrap-t confidence intervalsstep 1Set the simulation number $N_{boot}$ times ahead.step 2Compute the MLEs of σ and λ under the original censored sample $\underline{x}=\left(x_{1},x_{2},\cdots ,x_{m}\right)$, denoted as $\hat{\sigma }$ and $\hat{\lambda }$. (If we carry out a simulation study, we should first generate an adaptive progressive type II censored sample $\underline{x}=\left(x_{1},x_{2},\cdots ,x_{m}\right)$ from EHL(λ,σ) with T,n,m,R as the original sample.)step 3Generate a bootstrap sample ${\underline{x}}^{*}$ using $\hat{\sigma }$,$\hat{\lambda }$ and the same censoring pattern (n,m,T,R). Then, calculate the bootstrap MLEs ${\hat{\sigma }}^{*}$ and ${\hat{\lambda }}^{*}$ and their variances $Var({\hat{\sigma }}^{*})$ and $Var({\hat{\lambda }}^{*})$.step 4Calculate the t-statistics $\tilde{S_{1}}=\frac{{\hat{\sigma }}^{*}-\hat{\sigma }}{\sqrt{Var\left({\hat{\sigma }}^{*}\right)}}$ for ${\hat{\sigma }}^{*}$ and $\tilde{S_{2}}=\frac{{\hat{\lambda }}^{*}-\hat{\lambda }}{\sqrt{Var\left({\hat{\lambda }}^{*}\right)}}$ for ${\hat{\lambda }}^{*}$.step 5Repeat steps 2–3 $N_{boot}$ times to acquire a series of bootstrap t-statistics $\left({\tilde{S_{1}}}_{**}^{\left(1\right)},{\tilde{S_{1}}}_{**}^{\left(2\right)},\cdots ,{\tilde{S_{1}}}_{**}^{\left(N_{boot}\right)}\right)$ and $\left({\tilde{S_{2}}}_{**}^{\left(1\right)},{\tilde{S_{2}}}_{**}^{\left(2\right)},\cdots ,{\tilde{S_{2}}}_{**}^{\left(N_{boot}\right)}\right)$.step 6Arrange $\left({\tilde{S_{1}}}_{**}^{\left(1\right)},{\tilde{S_{1}}}_{**}^{\left(2\right)},\cdots ,{\tilde{S_{1}}}_{**}^{\left(N_{boot}\right)}\right)$ and $\left({\tilde{S_{2}}}_{**}^{\left(1\right)},{\tilde{S_{2}}}_{**}^{\left(2\right)},\cdots ,{\tilde{S_{2}}}_{**}^{\left(N_{boot}\right)}\right)$ in ascending order respectively and obtain $\left({\tilde{S_{1}}}_{**}^{\left[1\right]},{\tilde{S_{1}}}_{**}^{\;\left[2\right]},\cdots {\tilde{S_{1}}}_{**}^{\left[N_{boot}\right]}\right)$ and $\left({\tilde{S_{2}}}_{**}^{\left[1\right]},{\tilde{S_{2}}}_{**}^{\left[2\right]},\cdots ,{\tilde{S_{2}}}_{**}^{\left[N_{boot}\right]}\right)$.

## 4. Bayesian Estimation

In this section, we compute the Bayesian estimates of the quantities by using the Lindley method and the importance sampling procedure. Unlike classical statistics, Bayesian statistics treat quantities as random variables, which combines the prior information with observed information.

The option of prior distribution is a pivotal problem. Generally speaking, the conjugate prior distribution is the first choice due to its algebraic simplicity. However, it is very difficult to find such prior when both quantities σ and λ are unknown. The prior distribution is reasonable to keep the same form as (6). Suppose that $\sigma ∼IG\left(\gamma ,\delta \right)$ and $\lambda ∼Ga\left(\alpha ,\beta \right)$ and that these two priors are independent. The PDFs of their prior distributions correspond to

(19) $$\pi \left(\sigma \right)=\frac{\delta^{\gamma }}{\Gamma (\gamma )}\sigma^{-\gamma -1}e^{-\frac{\delta }{\sigma }},\;\gamma >0,\;\delta >0$$

(20) $$\pi \left(\lambda \right)=\frac{\beta^{\alpha }}{\Gamma (\alpha )}\lambda^{\alpha -1}e^{-\beta \lambda },\;\alpha >0,\;\beta >0.$$

The corresponding joint distribution is

(21) $$\pi \left(\sigma ,\lambda \right)=\frac{\delta^{\gamma }\beta^{\alpha }}{\Gamma (\gamma )\Gamma (\alpha )}\sigma^{-\gamma -1}\lambda^{\alpha -1}e^{-(\frac{\delta }{\sigma }+\beta \lambda )},$$

Given the sample $\underline{x}$, the posterior distribution $\pi (\sigma ,\lambda |\underline{x})$ can be written as

(22) $$\pi \left(\sigma ,\lambda |\underline{x}\right)=\frac{L\left(\underline{x}|\sigma ,\lambda \right)\pi \left(\sigma ,\lambda \right)}{\int_{0}^{\infty }\int_{0}^{\infty }L\left(\underline{x}|\sigma ,\lambda \right)\pi \left(\sigma ,\lambda \right)d\sigma d\lambda }.$$

### 4.1. Symmetric and Asymmetric Loss Functions

The loss function is employed to appraise the intensity of inconsistency between the estimation of the parameter and the true value. The squared error loss function is a symmetric loss function, which is applied in many areas. However, on the condition that overestimation causes greater loss compared with underestimation or vice versa, using a symmetric loss function is not suitable. Instead, the asymmetric loss function is employed to fix the problem. Therefore, we consider the Bayesian estimations under one symmetric loss function, namely the squared error loss function (SELF) as well as two asymmetric loss functions, namely the Linex Loss Function (LLF) and the General Entropy Loss Function (GELF) in this subsection.

#### 4.1.1. Squared Error Loss Function (SELF)

The squared error loss function is a symmetric loss function, which puts the overestimate and underestimate on the same level. It is the sum of squared distances between the target variable and the predicted value. The function corresponds to

(23) $$L_{SE}\left(\upsilon ,\hat{\upsilon }\right)={\left(\hat{\upsilon }-\upsilon \right)}^{2},$$

where $\hat{\upsilon }$ is the estimation of υ.

The Bayesian estimation of υ under SELF is given by

(24) $$\hat{\upsilon }=E_{\upsilon }\left(\upsilon |\underline{x}\right).$$

Then, for the unknown parameters σ and λ, the Bayesian estimates under SELF can be given directly as

(25) $${\hat{\sigma }}_{SE}=\int_{0}^{\infty }\int_{0}^{\infty }\sigma \pi \left(\sigma ,\lambda |\underline{x}\right)d\sigma d\lambda ,$$

(26) $${\hat{\lambda }}_{SE}=\int_{0}^{\infty }\int_{0}^{\infty }\lambda \pi \left(\sigma ,\lambda |\underline{x}\right)d\sigma d\lambda .$$

#### 4.1.2. Linex Loss Function (LLF)

The Linex function is a well-known asymmetric loss function. It is defined as

(27) $$L_{LL}\left(\upsilon ,\hat{\upsilon }\right)=e^{p(\hat{\upsilon }-\upsilon )}-p\left(\hat{\upsilon }-\upsilon \right)-1.$$

The size of *p* denotes the level of asymmetry and its sign represents the direction of asymmetry. For p<0, LLF alters exponentially in the negative direction and linearly in the positive direction, thus a negative bias has a more serious impact—while, for p>0, the positive error will be punished heavily. The larger the dimension of *p* is, the larger the punishment intensity is. When $\left|p\right|$ approaches 0, LLF is almost symmetric.

The Bayesian estimation of υ under LLF is written as

(28) $${\hat{\upsilon }}_{LL}=-\frac{1}{p}\ln E_{\upsilon }\left(e^{-p\upsilon }|\underline{x}\right).$$

Then, for unknown parameters σ and λ, the Bayesian estimates under LLF are

(29) $${\hat{\sigma }}_{LL}=-\frac{1}{p}\ln\left[\int_{0}^{\infty }\int_{0}^{\infty }e^{-p\sigma }\pi \left(\sigma ,\lambda |\underline{x}\right)d\sigma d\lambda \right],$$

(30) $${\hat{\lambda }}_{LL}=-\frac{1}{p}\ln\left[\int_{0}^{\infty }\int_{0}^{\infty }e^{-p\lambda }\pi \left(\sigma ,\lambda |\underline{x}\right)d\sigma d\lambda \right].$$

#### 4.1.3. General Entropy Loss Function (GELF)

The General Entropy loss function (GELF) is another noted asymmetric loss function, which is

(31) $$L_{GE}\left(\upsilon ,\hat{\upsilon }\right)={\left(\frac{\hat{\upsilon }}{\upsilon }\right)}^{q}-q\ln\frac{\hat{\upsilon }}{\upsilon }-1.$$

For q>0, the overestimation has a more serious impact compared with the underestimation, and vice versa. The Bayesian estimation of υ under GELF is derived:

(32) $${\hat{\upsilon }}_{GE}={\left[E_{\upsilon }\left(\upsilon^{-q}|\underline{x}\right)\right]}^{-\frac{1}{q}}.$$

Notably, when q=−1, the Bayesian estimation under GELF has the same value as that under SELF. The Bayesian estimates of σ and λ under GELF correspond to

(33) $${\hat{\sigma }}_{GE}={\left[\int_{0}^{\infty }\int_{0}^{\infty }\sigma^{-q}\pi \left(\sigma ,\lambda |\underline{x}\right)d\sigma d\lambda \right]}^{-\frac{1}{q}},$$

(34) $${\hat{\lambda }}_{GE}={\left[\int_{0}^{\infty }\int_{0}^{\infty }\lambda^{-q}\pi \left(\sigma ,\lambda |\underline{x}\right)d\sigma d\lambda \right]}^{-\frac{1}{q}}.$$

We can know that the Bayesian estimates of σ and λ are in the modality of a ratio of two complicated integrals and the specific and explicit forms cannot be represented without trouble. Thus, the Lindley method is employed to solve this problem.

### 4.2. Lindley Approximation Method

In this subsection, in order to compute the Bayesian estimates, we apply the Lindley approximation method. Let φ(σ,λ) denote any function about σ and λ, *l* denote the log-likelihood function and ρ(σ,λ)=lnπ(σ,λ). According to the [19], the Bayesian estimates can be expressed by the posterior expectation of φ(σ,λ)

(35) $$E\left[\varphi \left(\sigma ,\lambda \right)|\underline{x}\right]=\varphi \left(\hat{\sigma },\hat{\lambda }\right)+\rho_{1}A_{12}+\frac{1}{2}\left(A+l_{03}B_{21}+l_{30}B_{12}+l_{12}C_{21}+l_{21}C_{12}\right)+\rho_{2}A_{21},$$

where

$$\begin{array}{c} A=\sum_{i=1}^{2}\sum_{i=1}^{2}\varphi_{ij}b_{ij}\;l_{ij}=\frac{\partial^{i+j}l}{\partial \theta^{i}\partial \theta^{j}},i=3-j\;and\;i,j=0,1,2,3\\ \rho_{i}=\frac{\partial \rho }{\partial \theta_{i}},\varphi_{i}=\frac{\partial \rho }{\partial \theta_{i}},\varphi_{ij}=\frac{\partial^{2}\rho }{\partial \theta_{i}\partial \theta_{j}},b_{ij}=-{\left[l_{ij}\right]}^{-1},A_{ij}=\varphi b_{ii}+\varphi_{j}b_{ji},\\ B_{ij}=\left(\varphi_{i}b_{ii}+\varphi_{j}b_{ij}\right)b_{ii},C_{ij}=3\varphi_{i}b_{ii}b_{ij}+\varphi_{j}\left(b_{ii}b_{jj}+2b_{ij}^{2}\right). \end{array}$$

Here, $\theta =\left(\theta_{1},\theta_{2}\right)=\left(\sigma ,\lambda \right)$ and $b_{ij}$ denotes the (i,j)-th component of the covariance matrix. Then, the Bayesian estimates under three loss functions SELF, LLF, and GELF are derived.

#### 4.2.1. Squared Error Loss Function (SELF)

For σ, let φ(σ,λ)=σ; therefore,

(36) $$\varphi \left(\sigma ,\lambda \right)=\sigma ,\;\varphi_{1}=1,\;\varphi_{11}=\varphi_{12}=\varphi_{2}=\varphi_{21}=\varphi_{22}=0.$$

Then, the Bayesian estimate of σ under SELF is

(37) $${\hat{\sigma }}_{SE}=\hat{\sigma }+\frac{1}{2}\left[b_{11}^{2}l_{30}+3\;b_{11}b_{12}l_{21}+b_{11}b_{22}l_{12}+2b_{21}^{2}l_{12}+b_{21}b_{22}l_{03}\right]+\rho_{1}b_{11}+\rho_{2}b_{12}.$$

Similarly, for parameter λ, it is clear that φ(σ,λ)=λ, hence

(38) $$\varphi \left(\sigma ,\lambda \right)=\lambda ,\;\varphi_{2}=1,\;\varphi_{21}=\varphi_{22}=\varphi_{1}=\varphi_{11}=\varphi_{12}=0.$$

Then, the Bayesian estimate of λ under SELF can be written as

(39) $${\hat{\lambda }}_{SE}=\hat{\lambda }+\frac{1}{2}\left[b_{11}b_{12}l_{30}+b_{11}b_{22}l_{21}+2b_{12}^{2}l_{12}+3b_{21}b_{22}l_{12}+b_{22}^{2}l_{03}\right]+\rho_{1}b_{21}+\rho_{2}b_{22}.$$

#### 4.2.2. Linex Loss Function (LLF)

For σ, we take $\varphi \left(\sigma ,\lambda \right)=e^{-p\sigma }$, hence

(40) $$\varphi_{1}=-pe^{-p\sigma },\;\varphi_{11}=p^{2}e^{-p\sigma },\;\varphi_{2}=\varphi_{12}=\varphi_{21}=\varphi_{22}=0.$$

The Bayesian estimate of σ under LLF is derived as

(41) $$\begin{array}{ccc} {\hat{\sigma }}_{LL} & = & -\frac{1}{p}\ln\{e^{-p\hat{\sigma }}+\frac{1}{2}\varphi_{11}b_{11}+\frac{1}{2}\varphi_{1}[b_{11}^{2}l_{30}+3b_{11}b_{12}l_{21}\\ & & +b_{11}b_{22}l_{12}+2b_{21}^{2}l_{12}+b_{21}b_{22}l_{03}]+\varphi_{1}\left(\rho_{1}b_{11}+\rho_{2}b_{12}\right)\}. \end{array}$$

Similarly, for the parameter λ, let $\varphi \left(\sigma ,\lambda \right)=e^{-p\lambda }$, hence

(42) $$\varphi_{2}=-pe^{-p\lambda },\;\varphi_{22}=p^{2}e^{-p\lambda },\;\varphi_{1}=\varphi_{11}=\varphi_{12}=\varphi_{21}=0.$$

The Bayesian estimate of λ under LLF can be written as

(43) $$\begin{array}{ccc} {\hat{\lambda }}_{LL} & = & -\frac{1}{p}\ln\{e^{-p\hat{\lambda }}+\frac{1}{2}\varphi_{22}b_{22}+\frac{1}{2}\varphi_{2}[b_{22}^{2}l_{03}+3b_{22}b_{21}l_{12}\\ & & +b_{11}b_{22}l_{21}+2b_{12}^{2}l_{21}+b_{12}b_{11}l_{30}]+\varphi_{2}\left(\rho_{1}b_{21}+\rho_{2}b_{22}\right)\}. \end{array}$$

#### 4.2.3. General Entropy Loss Function (GELF)

For parameter σ, let $\varphi \left(\sigma ,\lambda \right)=\sigma^{-q}$, hence

(44) $$\varphi_{1}=-q\sigma^{-q-1},\;\varphi_{11}=q\left(q+1\right)\sigma^{-q-2},\;\varphi_{2}=\varphi_{12}=\varphi_{21}=\varphi_{22}=0.$$

The Bayesian estimate of σ under GELF can be written as

(45) $$\begin{array}{ccc} {\hat{\sigma }}_{GE} & = & \{{\hat{\sigma }}^{-q}+\frac{1}{2}\varphi_{11}b_{11}+\frac{1}{2}\varphi_{1}[b_{11}^{2}l_{30}+3b_{11}b_{12}l_{21}+b_{11}b_{12}l_{12}\\ & & +2b_{21}^{2}l_{12}+b_{21}b_{22}l_{03}{]+\varphi_{2}\left(\rho_{1}b_{11}+\rho_{2}b_{12}\right)\}}^{\frac{1}{q}}. \end{array}$$

Similarly, for parameter λ,   , it is clear that $\varphi \left(\sigma ,\lambda \right)=\lambda^{-q}$, hence

(46) $$\varphi_{2}=-q\lambda^{-q-1},\;\varphi_{22}=q\left(q+1\right)\lambda^{-q-2},\;\varphi_{1}=\varphi_{11}=\varphi_{21}=\varphi_{12}=0.$$

The Bayesian estimate of λ under GELF can be written as

(47) $$\begin{array}{ccc} {\hat{\sigma }}_{GE} & = & \{{\hat{\lambda }}^{-q}+\frac{1}{2}\varphi_{22}b_{22}+\frac{1}{2}\varphi_{2}[b_{22}^{2}l_{03}+3b_{22}b_{21}l_{12}+b_{11}b_{22}l_{21}\\ & & +2b_{12}^{2}l_{21}+b_{12}b_{11}l_{30}{]+\varphi_{2}\left(\rho_{1}b_{21}+\rho_{2}b_{22}\right)\}}^{\frac{1}{q}}. \end{array}$$

Though the Lindley approximation is effective to obtain point estimations by estimating the ratio of integrals, it can not provide credible intervals of the unknown parameters. Therefore, the importance sampling method is adopted to gain not only point estimation but also credible intervals.

### 4.3. Importance Sampling Procedure

The importance sampling procedure is an extension to the Monte Carlo method, which can greatly reduce the number of sample points drawn in the simulation, and is widely used in the reliability analysis of various models. From (6) and (21), the joint posterior distribution is derived by

(48) $$\begin{array}{ccc} \pi (\sigma ,\lambda |\underline{x}) & \propto & \frac{\delta^{\gamma }\beta^{\alpha }}{\Gamma (\gamma )\Gamma (\alpha )}\sigma^{-m-\gamma -1}\lambda^{m+\alpha -1}e^{-\lambda \sum_{i=1}^{m}\ln\frac{1+e^{-\frac{x_{i}}{\sigma }}}{1-e^{-\frac{x_{i}}{\sigma }}}-\frac{1}{\sigma }\sum_{i=1}^{m}x_{i}-\frac{\delta }{\sigma }-\beta \lambda }\\ & & \times \prod_{i=1}^{m}\frac{1}{1-e^{-\frac{2x_{i}}{\sigma }}}\prod_{i=1}^{J}{\left[1-{\left(\frac{1-e^{-\frac{x_{i}}{\sigma }}}{1+e^{-\frac{x_{i}}{\sigma }}}\right)}^{\lambda }\right]}^{R_{i}}{\left[1-{\left(\frac{1-e^{-\frac{x_{m}}{\sigma }}}{1+e^{-\frac{x_{m}}{\sigma }}}\right)}^{\lambda }\right]}^{n-m-\sum_{i=1}^{J}R_{i}}\\ & \propto & h_{1}\left(\sigma \right)h_{2}\left(\lambda |\sigma \right)h_{3}\left(\sigma ,\lambda \right), \end{array}$$

where

(49) $$h_{1}\left(\sigma \right)=\frac{{\left(\delta +\sum_{i=1}^{m}x_{i}\right)}^{\gamma +m}}{\Gamma (\gamma +m)}\sigma^{-(\gamma +m+1)}e^{-\frac{\delta +\sum_{i=1}^{m}}{\sigma }},$$

(50) $$h_{2}\left(\lambda |\sigma \right)=\frac{{\left[\beta +\sum_{i=1}^{m}\ln\frac{1+e^{-\frac{x_{i}}{\sigma }}}{1-e^{-\frac{x_{i}}{\sigma }}}\right]}^{\alpha +m}}{\Gamma (\alpha +m)}\lambda^{\alpha +m-1}e^{-\lambda (\beta +\sum_{i=1}^{m}\ln\frac{1-e^{-\frac{x_{i}}{\sigma }}}{1+e^{-\frac{x_{i}}{\sigma }}})},$$

(51) $$h_{3}\left(\sigma ,\lambda \right)=\frac{1}{{\left[\beta +\sum_{i=1}^{m}\ln\frac{1+e^{-\frac{x_{i}}{\sigma }}}{1-e^{-\frac{x_{i}}{\sigma }}}\right]}^{\alpha +m}}\prod_{i=1}^{m}\frac{1}{1-e^{-\frac{2x_{i}}{\sigma }}}\prod_{i=1}^{J}{\left[1-{\left(\frac{1-e^{-\frac{x_{i}}{\sigma }}}{1+e^{-\frac{x_{i}}{\sigma }}}\right)}^{\lambda }\right]}^{R_{i}}{\left[1-{\left(\frac{1-e^{-\frac{x_{m}}{\sigma }}}{1+e^{-\frac{x_{m}}{\sigma }}}\right)}^{\lambda }\right]}^{n-m-\sum_{i=1}^{J}R_{i}}.$$

It is clear that $h_{1}\left(\sigma \right)$ is the PDF of an inverse Gamma distribution while $h_{2}\left(\lambda \right)$ is the PDF of a Gamma distribution.

Therefore, the Bayesian estimation of $\varphi \left(\sigma ,\lambda \right)$ is acquired by the following steps:

1. Generate σ from $IG_{\sigma }\left(\gamma +m,\delta +\sum_{i=1}^{m}x_{i}\right)$.
2. On the basis of step 1, generate λ from $Ga_{\lambda |\sigma }\left(m+\alpha ,\sum_{i=1}^{m}\ln\frac{1+e^{-\frac{x_{i}}{\sigma }}}{1-e^{-\frac{x_{i}}{\sigma }}}+\beta \right)$.
3. Repeat step 1 and step 2 *M* times and produce a series of samples.
4. The Bayesian estimate of φ(σ,λ) is calculated by
  (52) $$\hat{\varphi }\left(\sigma ,\lambda \right)=\frac{\sum_{i=1}^{M}\varphi \left(\sigma_{i},\lambda_{i}\right)h_{3}\left(\sigma_{i},\lambda_{i}\right)}{\sum_{i=1}^{M}h_{3}\left(\sigma_{i},\lambda_{i}\right)}.$$

Therefore, the Bayesian estimate of the unknown parameter σ and λ is derived by

$$\hat{\sigma }=\frac{\sum_{i=1}^{M}\sigma_{i}h_{3}\left(\sigma_{i},\lambda_{i}\right)}{\sum_{i=1}^{M}h_{3}\left(\sigma_{i},\lambda_{i}\right)},$$

$$\hat{\lambda }=\frac{\sum_{i=1}^{M}\lambda_{i}h_{3}\left(\sigma_{i},\lambda_{i}\right)}{\sum_{i=1}^{M}h_{3}\left(\sigma_{i},\lambda_{i}\right)}.$$

Let

(53) $$h_{3i}\left(\sigma_{i},\lambda_{i}\right)=\frac{h_{3}\left(\sigma_{i},\lambda_{i}\right)}{\sum_{i=1}^{M}h_{3}\left(\sigma_{i},\lambda_{i}\right)}.$$

For the sake of simplicity, $h_{3i}\left(\sigma_{i},\lambda_{i}\right)$ is denoted as $h_{3i}$. Then, we sort $\left\{\sigma_{1},\sigma_{2}\ldots ,\sigma_{M}\right\}$ in ascending order as $\left\{\sigma_{\left(1\right)},\sigma_{\left(2\right)}\ldots ,\sigma_{\left(M\right)}\right\}$. In addition, we combine $h_{3i}$ and $\sigma_{i}$ together as $\{\left(\sigma_{\left(1\right)},h_{3\left(1\right)}\right),$$\left(\sigma_{\left(2\right)},h_{3\left(2\right)}\right),\ldots \left(\sigma_{\left(M\right)},h_{3\left(M\right)}\right)\}$. The HPD credible interval is established based on the estimate ${\hat{\sigma }}_{p}=\sigma_{\left(g_{p}\right)}$, where $g_{p}$ is an integer that satisfies

(54) $$\sum_{i=1}^{g_{p}}h_{3(i)}\leq p\leq \sum_{i=1}^{g_{p}+1}h_{3(i)}.$$

Hence, the 100%(1−α) credible interval can be represented as $\left({\hat{\sigma }}_{\zeta },{\hat{\sigma }}_{\zeta +1-\alpha }\right)$, $\zeta =h_{3(1)},h_{3(1)}+h_{3(2)},\cdots ,\sum_{i=1}^{g_{p}}h_{3(i)}$. Therefore, the HPD credible for σ is obtained by $\left({\hat{\sigma }}_{\zeta^{*}},{\hat{\sigma }}_{\zeta^{*}+1-\alpha }\right)$. Note that ${\hat{\sigma }}_{\zeta^{*}+1-\alpha }-{\hat{\sigma }}_{\zeta^{*}}\leq {\hat{\sigma }}_{\zeta +1-\alpha }-{\hat{\sigma }}_{\zeta }$ for all ζ.

## 5. Simulation

Plenty of simulation experiments are carried out to appraise the performance of our estimations by Monte Carlo simulations. Here, the R software is employed for all the simulations. The point estimation is evaluated by the mean square error (MSE) and estimation value (VALUE), while the interval estimation is assessed based on the coverage rate (CR) and interval mean length (ML). For point estimation, smaller mean square error and closer estimation value suggest better performance of estimation. In addition, for interval estimation, the higher the coverage rate is and the narrower the interval mean length is, the better the estimation is.

First of all, adaptive type II progressive censored data from an exponentiated half-logistic distribution should be generated. The algorithm for generating adaptive Type II progressive censored data from a general distribution can be obtained in [3]. The algorithm to generate the censored data is listed in Algorithm 3.
**Algorithm 3:** Generating adaptive type II progressive censored data from EHL(λ,σ).1.Generate a Type II progressive censored sample from an exponentiated half-logistic distribution EHL(λ,σ) with initial values of $\left(R_{1},R_{2},\cdots ,R_{m}\right)$ and T,n,m:(a)generate independent random variables $U_{1},U_{2},\cdots ,U_{m}$ from the uniform distribution U(0,1).(b)Let $V_{i}=U_{i}^{\frac{1}{i+\sum_{j=m-i+1}^{m}R_{i}}}$, i=1,2,⋯,m.(c)Let $W_{i}=1-V_{m}V_{m-1}\cdots \;V_{m-i+1}$, i=1,2,⋯,m.(d)For certain σ and λ, let $X_{i}=F^{-1}\left(W_{i}\right)$. Then, $X=(X_{1},X_{2},\cdots ,\;X_{m})$ is the Type II progressive censored sample from EHL(λ,σ).2.Confirm the value of *J*, and abandon the sample $X_{J+2},\cdots ,X_{m}$.3.Generate the first m−J−1 order statistics from a truncated distribution $\frac{f(x)}{\left[1-F\left(x_{J+1}\right)\right]}$ with sample size $n-(\sum_{i=1}^{J}R_{i}+J+1)$ as $X_{J+2},X_{J+3},\cdots ,X_{m}$.

In order to carry out simulations, we set σ=1.5 and λ=1. For comparison purposes, we consider T=2,4 and (n,m)=(30,20),(30,25),(50,40),(50,45),(80,60),(80,70). For all the combinations of sample size and time *T*, two different censoring schemes (CS) are chosen:

Scheme I (Sch I): $R_{1}=n-m,R_{k}=0,k=2,3,\cdots ,m$.
Scheme II (Sch II): $R_{1}=R_{2}=\cdots =R_{n-m}=1,R_{k}=0,k>n-m$.

In addition, the specific diverse censoring schemes conceived for the simulation are listed in Table 1.

For simplicity, we abbreviate the censoring schemes. For example, (1, 1, 1, 0, 0, 0, 0) is represented as (1*3, 0*4). In each case, the simulation is repeated 3000 times. Then, the associated MSEs and VALUEs with the point estimation and the related coverage rates and mean lengths with the interval estimation can be acquired through Monte Carlo simulations using R software.

For maximum likelihood estimation, the L-BFGS-B method is used and the simulation results are put into Table A1. In Bayesian estimation, we employ not only non-informative priors (non-infor) but also informative priors (infor). For the non-informative priors, we set α=β=γ=δ=0.0001. Then, for the informative priors, we should first determine the hyper-parameters for Bayesian estimation. Generally speaking, the actual value of the parameter is usually considered as the expectation of the prior distribution. However, due to the complexity and interactive influence of the two prior distributions, the optimal value can not be found directly. Thus, we adopt a genetic algorithm and simulated annealing algorithm to determine the optimal hyper-parameters and the results are: γ=4.5,δ=7.5,α=4.5,β=4.5. To get Bayesian point estimation, the Lindley method and the importance sampling method are employed. Three loss functions are adopted separately for comparison purposes. The parameter *p* of LLF is set to 0.5 and 1 and the parameter *q* of GELF is set to −0.5 and 0.5.

The informative Bayes method uses minimization of loss functions, and such minimizations can only be performed if the true parameter values are known. Hence, informative Bayes can only be seen as a reference, or an oracle method.

The results are presented in Table A2, Table A3, Table A4, Table A5, Table A6, Table A7, Table A8 and Table A9. In addition, the mean length and coverage rate of asymptotic confidence intervals, boot-t intervals, boot-p intervals, and HPD intervals at 95% confidence/credible level are also shown in Table A10 and Table A11.

**Figure 3:**
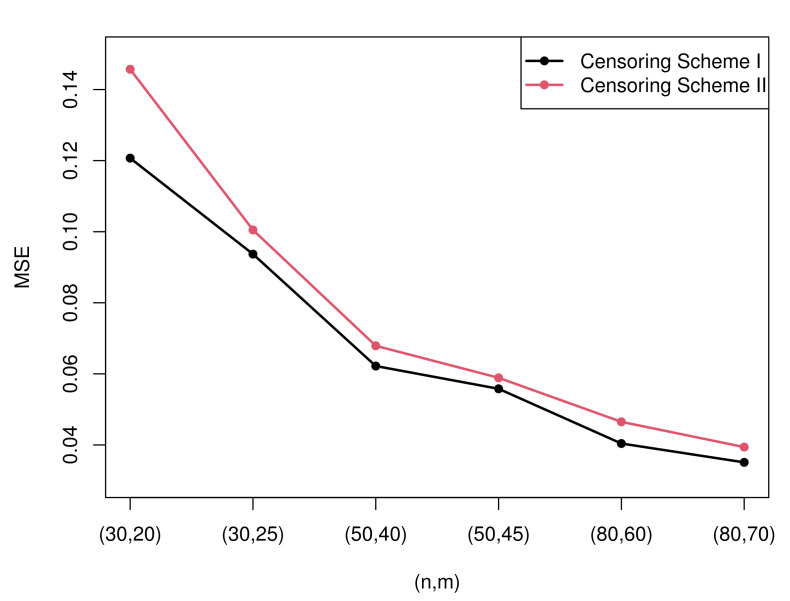
The MSEs of the MLEs of parameter σ under two censoring schemes.

**Figure 4:**
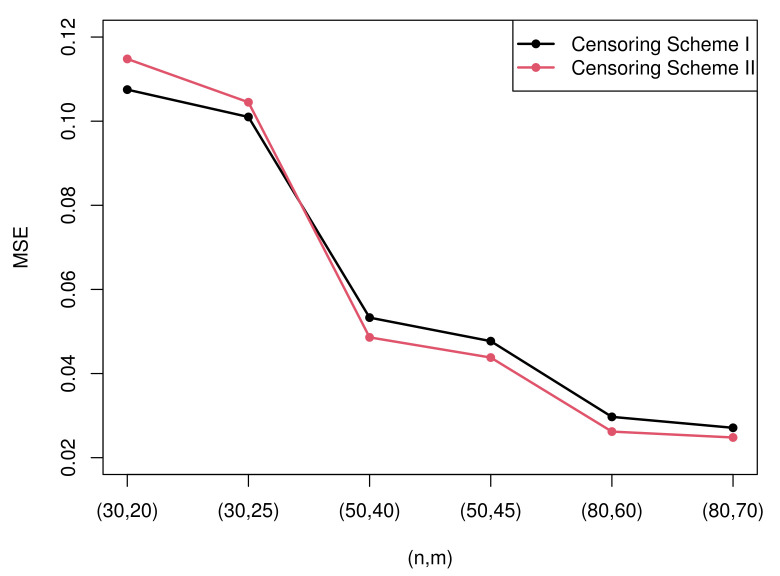
The MSEs of the MLEs of parameter λ under two censoring schemes.

**Figure 5:**
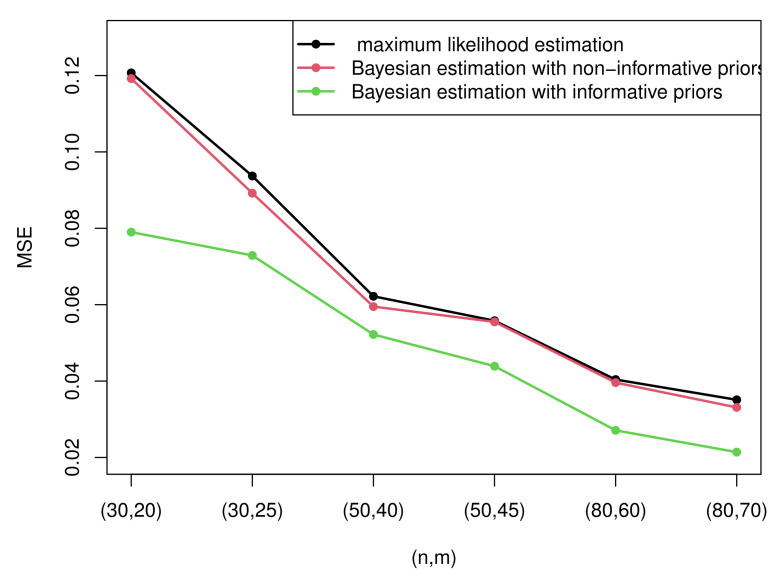
The MSEs of MLEs and Bayesian estimates with non-informative and informative priors of parameter σ.

**Figure 6:**
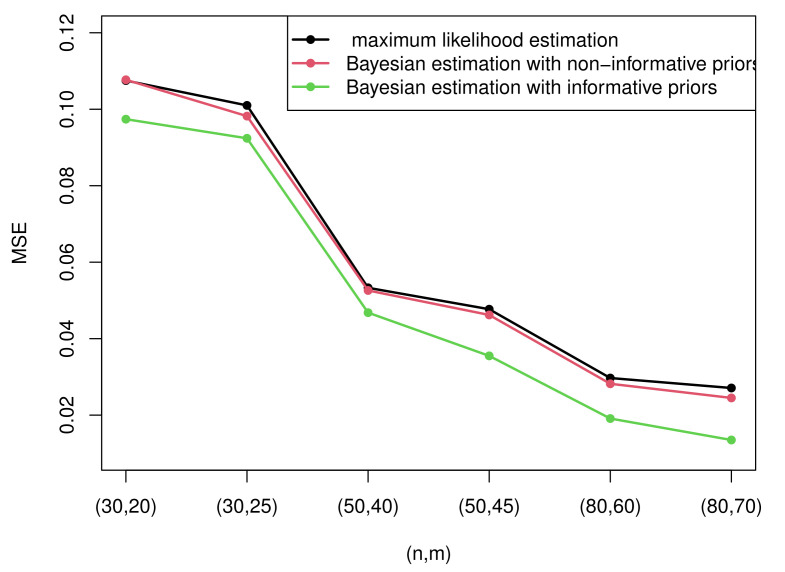
The MSEs of MLEs and Bayesian estimates with non-informative and informative priors of parameter σ.

Due to the excessive amount of tables, it is not easy for readers to find rules of the estimation. Therefore, some figures which present the most representative simulation results are made to show the rules more intuitively. Figure 3 and Figure 4 present the MSEs of the maximum likelihood estimates of the two parameters under censoring scheme I and censoring scheme II when T=2. Figure 5 and Figure 6 compare the MSEs of maximum likelihood estimates with the Bayesian estimates with non-informative and informative priors obtained by importance sampling under censoring scheme I and T=2.

From Table A1, we can draw that

(1) All the estimation values are generally inclined to approach the true value, and MSEs tend to decrease as the sample size *n* or observed numbers *m* or the value of m/n increases. The rules of the MSEs can be easily obtained from Figure 3 and Figure 4.
(2) The MLEs of λ perform better than the MLEs of σ according to the MSE. However, the estimation values of σ are closer to the true value compared with those of λ.
(3) Diverse censoring schemes show a regular mode in terms of MSE. From the Figure 3 and Figure 4, we can know that, when σ is considered, Sch I performs better than Sch II in all cases, yet when λ is considered, Sch II is more effective than Sch I except the case of n=30.
(4) There is no observed specific pattern with the change of *T*. It is apprehensible because the observed data may remain unaltered when *T* changes.

From Table A2, Table A3, Table A4, Table A5, Table A6, Table A7, Table A8 and Table A9, we can find that

(1) Generally, the Bayesian estimates under three loss functions with informative priors are more accurate contrasted with MLEs in terms of MSE in all cases. This rule can be intuitively summarized from Figure 5 and Figure 6. This is because the Bayesian method not only considers the data but also takes the prior information of unknown parameters into account. In addition, the importance sampling procedure outperforms the Lindley method.
(2) From Figure 5 and Figure 6, it is clear that the performance of the Bayesian estimates with non-informative priors is almost similar to MLEs under all circumstances. This is because we have no information with respect to the unknown parameters. In other words, it only takes the data into account. Thus, it is reasonable that the results are analogous to MLEs.
(3) The Bayesian estimates under GELF are superior compared with those under SELF and LLF. For LLF, Bayesian estimates under p=1 are better than those under p=0.5 for the parameter λ, while choosing p=0.5 is better than p=1 for the estimate of σ. For GELF, take the fact that both q=−0.5 and q=0.5 are satisfactory and perform well. On the whole, the Bayesian estimates under GELF using the importance sampling procedure are the most effective as they possess the minimal MSEs and the closest estimation values.
(4) When σ is considered, Sch I performs better than Sch II except when n=50, yet when λ is taken into account, Sch II is superior compared with Sch I in most cases.

From Table A10 and Table A11, we can draw these conclusions

(1) The mean lengths of all the intervals become narrower as *n* and *m* increase, and this pattern holds for both σ and λ. In addition, the coverage rate of intervals of σ is higher while the coverage rate of intervals of λ is stable with the increase of *m* and *n*.
(2) The HPD credible intervals and boot-t intervals perform better contrasted by asymptotic confidence intervals due to narrower mean length and higher coverage rate. In addition, the HPD credible intervals possess the narrowest mean length while the boot-t intervals have the highest coverage rate.
(3) The results of the two parameters’ intervals have no obvious connection with different censoring schemes.

## 6. Real Data Analysis

An authentic dataset is analyzed for expository intention by employing the methods mentioned above in this section. The dataset was initially from [20] and further employed by [21,22]. The complete data set describes log times to the breakdown of an insulating fluid testing experiment and is presented in Table 2.

At the beginning, we should consider the problem whether the distribution EHL$\left(\lambda ,\sigma \right)$ fits the data set well. The fitting effect of exponentiated half-logistic distribution and Half Logistic distribution with the CDF $F\left(x\right)=\frac{1-e^{-\frac{x-\lambda }{\sigma }}}{1+e^{-\frac{x-\lambda }{\sigma }}}$ is compared. The criteria employed for examining the goodness of fit include the negative log-likelihood function (−lnL), Kolmogorov–Smirnov (K-S) statistics with its *p*-value, Bayesian Information Criterion (BIC), and Akaike Information Criterion (AIC). The definitions are:

$$AIC=2\times \left(d-\ln L\right),$$

BIC=d×lnn−2×lnL,

where *d* is the number of parameters, *L* is the maximized value of the likelihood function, and *n* denotes the total number of observed values.

The results of the K-S, *p*-value, AIC, BIC, and −lnL of the two distributions are listed in Table 3. Obviously, exponentiated half-logistic distribution fits the model better since it has lower K-S, AIC, BIC, −lnL statistics, and higher *p*-value. Then, we can analyze this data on the basis of our model.

We set n=16,m=12 and $T=\frac{3}{2},$ 2. The two different censoring schemes are (4,0∗11) and (1∗4,0∗8). Table 4 presents the specific adaptive type II censoring data under different schemes based on the data set.

The point estimations for σ and λ are presented in Table 5 and Table 6. For Bayesian estimation, since we have no informative prior, a non-informative prior is applied, namely α=β=γ=δ=0.0001. Three loss functions are considered, and we still use the parameters in the previous simulation. At the same time, 95% ACIs, boot-p, boot-t, and HPD intervals are established, while Table 7 and Table 8 display the corresponding results. Let Lower denote the lower bound and Upper denote the upper bound.

From Table 5, Table 6, Table 7 and Table 8, the following conclusions are drawn:

(1) The estimates of parameter σ using the Lindley method generally tend to be larger than those gained by the importance sampling procedure.
(2) The estimates under the first censoring scheme are closer to the MLEs under the full sample, and the estimations using the Lindley method are more effective than those obtained by the importance sampling.
(3) The results are relatively close between T=1.5 and T=2 when using the first censoring scheme because the observed data remain unaltered when the T is increasing.
(4) The HPD credible intervals have the narrowest mean length among all the intervals while the ACIs possess the longest mean length.
(5) The results of the two parameters’ intervals have no obvious connection with different censoring schemes.

## 7. Conclusions

In this manuscript, classical and Bayesian inference for exponentiated half-logistic distribution under adaptive Type II progressive censoring is considered. The maximum likelihood estimates are derived through the Newton–Raphson algorithm. Bayesian estimation under three loss functions is also considered and the estimates are derived through importance sampling and the Lindley method. Meanwhile, we establish the confidence and credible intervals of σ and λ and contrast them with each other. Asymptotic confidence intervals are constructed based on observed and expected Fisher information matrices. In order to tackle the problem of small sample size, boot-p and boot-t intervals are computed.

In the simulation section, estimation values and mean squared values are calculated to test the performance of the point estimation while mean lengths and coverage rates are considered for the interval estimation. According to the simulation results, it is clear that the Bayesian estimation which possesses suitable informative priors performs better than MLEs under all circumstances. In more detail, the Bayesian estimations under GELF perform best among all the estimations and the importance sampling procedure makes more sense than Lindley approximation. In addition, when it comes to interval estimation, boot-t and boot-p intervals perform better in the case of a small sample size than asymptotic confidence intervals. In addition, HPD credible intervals generally possess the shortest mean length while boot-t intervals have the highest coverage rate compared with other intervals.

Exponentiated half-logistic distribution under adaptive Type II progressive censoring is significant and practical due to the flexibility of the censoring scheme and the superior features of distribution. Furthermore, the competing risks and accelerated life test can be explored in the research field. In brief, carrying out further research on this model has great potential for survival and reliability analysis.

## Figures and Tables

**Table 1:** Different censoring schemes.

*T*	*n*	*m*	CS	*T*	*n*	*m*	CS
2	30	20	(10, 0*19)	4	30	20	(10, 0*19)
			(1*10, 0*10)				(1*10, 0*10)
		25	(5, 0*24)			25	(5, 0*24)
			(1*5, 0*20)				(1*5, 0*20)
	50	40	(10, 0*39)		50	40	(10, 0*39)
			(1*10, 0*30)				(1*10, 0*30)
		45	(5, 0*44)			45	(5, 0*44)
			(1*5, 0*40)				(1*5, 0*40)
	80	60	(20, 0*59)		80	60	(20, 0*59)
			(1*20, 0*40)				(1*20, 0*40)
		70	(10, 0*69)			70	(10, 0*69)
			(1*10, 0*60)				(1*10, 0*60)

**Table 2:** Real data set.

0.270027	1.02245	1.15057	1.42311	1.54116	1.57898	1.8718	1.9947
2.08069	2.11263	2.48989	3.45789	3.48187	3.52371	3.60305	4.28895

**Table 3:** The fitting results of the two distributions.

	λ	σ	−lnL	AIC	BIC	K-S Statistic	*p*-Value
HL	1.0023	0.6536	27.0313	56.6609	56.2061	0.2659	0.3749
EHL	2.4309	0.9639	24.4488	52.8976	54.4428	0.1836	0.5906

**Table 4:** Adaptive progressive type II censoring data under different schemes.

Scheme	Censored Data
(4, 0*11), T=1.5	0.270027, 1.57898, 1.8718, 1.9947, 2.08089, 2.11263
	2.48989, 3.45789, 3.481865, 3.52371, 3.60305, 4.28895
(4, 0*11), T=2	0.270027, 1.57898, 1.8718, 1.9947, 2.08089, 2.11263
	2.48989, 3.45789, 3.481865, 3.52371, 3.60305, 4.28895
(1*4, 0*8), T=1.5	0.270027, 1.15057, 1.54116, 1.57898, 1.8718, 1.9947
	2.08089, 2.11263, 2.48989, 3.45789, 3.481865, 3.52371
(1*4, 0*8), T=2	0.270027, 1.15057, 1.54116, 1.8718, 2.08089, 2.11263
	2.48989, 3.45789, 3.48187, 3.52371, 3.60305, 4.28895

**Table 5:** The MLEs and Bayesian estimates of σ under SELF, LLF, and GELF by the Lindley approximation and the importance sampling.

*T*	*R*	MLE	SELF	LLF		GELF		Method
				*p* = $\frac{1}{2}$	*p* = 1	*p* = −$\frac{1}{2}$	*p* = $\frac{1}{2}$	
1.5	(4, 0*11)	1.1958	1.1285	1.1104	1.0942	1.1134	1.0875	Lindley
			1.2577	1.3180	1.0408	1.0662	1.2887	Importance sampling
	(1*4, 0*11)	1.2014	1.0794	1.0626	1.0483	1.0654	1.0429	Lindley
			1.2346	1.1696	1.1458	1.1257	1.1306	Importance sampling
2	(4, 0*11)	1.1958	1.0340	1.0197	1.0061	1.0206	0.9959	Lindley
			1.3057	1.0047	1.2387	1.2787	0.9836	Importance sampling
	(1*4, 0*11)	1.2326	0.9577	0.9451	0.9333	0.9451	0.9223	Lindley
			1.3420	1.2877	1.2147	1.1860	1.3209	Importance sampling

**Table 6:** The MLEs and Bayesian estimates of λ under SELF, LLF, and GELF by the Lindley approximation and the importance sampling.

*T*	*R*	MLE	SELF	LLF		GELF		Method
				*p* = $\frac{1}{2}$	*p* = 1	*p* = −$\frac{1}{2}$	*p* = $\frac{1}{2}$	
1.5	(4, 0*11)	2.4364	2.3591	2.3883	2.3182	2.2932	2.3234	Lindley
			2.4817	2.2303	2.3475	2.5060	2.3174	Importance sampling
	(1*4, 0*11)	2.3748	2.5351	2.3908	2.1896	2.5062	2.3240	Lindley
			2.5865	2.3003	2.4913	2.4253	2.4157	Importance sampling
2	(4, 0*11)	2.4364	2.5786	2.3282	2.1437	2.4798	2.2910	Lindley
			2.1038	2.1082	1.8596	2.0381	2.0456	Importance sampling
	(1*4, 0*11)	2.3820	2.7732	2.5294	2.3202	2.7069	2.5050	Lindley
			2.5353	2.1758	2.5118	2.4388	2.4546	Importance sampling

**Table 7:** The four intervals for σ at the 95% confidence/credible level.

*T*	*R*	ACI		boot-p		boot-t		HPD	
		Lower	Upper	Lower	Upper	Lower	Upper	Lower	Upper
1.5	(4, 0*11)	0.6568	1.7348	0.6645	1.7800	0.8590	1.6932	0.7425	1.6066
	(1*4, 0*11)	0.6243	1.7785	0.6399	2.0750	0.7176	1.7313	0.8460	1.7601
2	(4, 0*11)	0.6568	1.7348	0.6001	1.7640	0.6955	1.7824	0.6969	1.6236
	(1*4, 0*11)	0.6243	1.7785	0.6340	1.9732	0.8295	1.9510	0.9078	1.4993

**Table 8:** The four intervals for λ at the 95% confidence/credible level.

*T*	*R*	ACI		boot-p		boot-t		HPD	
		Lower	Upper	Lower	Upper	Lower	Upper	Lower	Upper
1.5	(4, 0*11)	0.5197	4.3530	1.3134	3.8975	0.8036	3.2435	1.0744	3.3039
	(1*4, 0*11)	0.5143	4.0354	1.2444	4.3882	1.0538	3.8101	0.7514	3.2800
2	(4, 0*11)	0.5197	4.3530	1.3085	4.0165	1.5664	4.3491	0.8151	3.4981
	(1*4, 0*11)	0.5143	4.0354	1.3628	3.8839	0.4289	3.2993	1.0254	3.7863

## Data Availability

The data presented in this study are openly available in [20].

## Appendix A. The Simulation Results of MLEs

**Table A1:** The simulation results of MLEs for σ and λ.

*T*	*n*	*m*	Sch	σ		λ	
				VALUE	MSE	VALUE	MSE
2	30	20	I	1.4694	0.1207	1.1028	0.1075
			II	1.4661	0.1457	1.1075	0.1138
		25	I	1.4667	0.0937	1.1024	0.1010
			II	1.4754	0.1005	1.1006	0.1025
	50	40	I	1.4787	0.0622	1.0607	0.0533
			II	1.4792	0.0649	1.0565	0.0496
		45	I	1.4832	0.0558	1.0580	0.0477
			II	1.4816	0.0559	1.0553	0.0458
	80	60	I	1.4898	0.0404	1.0403	0.0297
			II	1.4858	0.0425	1.0341	0.0272
		70	I	1.4858	0.0351	1.0348	0.0271
			II	1.4892	0.0364	1.0337	0.0258
4	30	20	I	1.4651	0.1260	1.1133	0.1140
			II	1.4573	0.1316	1.1152	0.1170
		25	I	1.4655	0.0990	1.1063	0.1039
			II	1.4661	0.1025	1.1049	0.1057
	50	40	I	1.4772	0.0583	1.0569	0.0503
			II	1.4857	0.0655	1.0500	0.0454
		45	I	1.4823	0.0572	1.0518	0.0474
			II	1.4805	0.0580	1.0542	0.0456
	80	60	I	1.4904	0.0419	1.0342	0.0305
			II	1.4912	0.0445	1.0303	0.0288
		70	I	1.4843	0.0352	1.0342	0.0266
			II	1.4884	0.0369	1.0336	0.0264

## Appendix B. The Simulation Results of Bayesian Estimates with Non-Informative Priors

**Table A2:** The results of Bayesian estimates with non-informative priors for σ using the Lindley method.

*T*	*n*	*m*	${\hat{\sigma }}_{\mathrm{SE}}$		${\hat{\sigma }}_{\mathrm{LL}}$				${\hat{\sigma }}_{\mathrm{GE}}$			
					$p=\frac{1}{2}$		p=1		$q=-\frac{1}{2}$		$q=\frac{1}{2}$	
			**VALUE**	**MSE**	**VALUE**	**MSE**	**VALUE**	**MSE**	**VALUE**	**MSE**	**VALUE**	**MSE**
2	30	20	1.5300	0.1187	1.5303	0.1183	1.5301	0.1201	1.5309	0.1156	1.5296	0.1117
			1.5328	0.1415	1.5347	0.1376	1.5335	0.1397	1.5333	0.1386	1.5329	0.1397
		25	1.5289	0.0926	1.5336	0.0891	1.5323	0.0916	1.5327	0.0917	1.5330	0.0864
			1.5346	0.1003	1.5247	0.1010	1.5244	0.9938	1.5242	0.0971	1.5242	0.0965
	50	40	1.5209	0.0596	1.5221	0.0612	1.5209	0.0603	1.5210	0.0622	1.5201	0.0572
			1.5206	0.0636	1.5210	0.0659	1.5200	0.0632	1.5199	0.0657	1.5198	0.0603
		45	1.5202	0.0541	1.5175	0.0546	1.5167	0.0559	1.5164	0.0504	1.5162	0.0525
			1.5195	0.0563	1.5194	0.0548	1.5181	0.0560	1.5174	0.0535	1.5183	0.0504
	80	60	1.5092	0.0402	1.5105	0.0401	1.5098	0.0397	1.5101	0.0353	1.5092	0.0285
			1.5136	0.0438	1.5143	0.0431	1.5138	0.0419	1.5137	0.0415	1.5131	0.0404
		70	1.5126	0.0341	1.5148	0.0339	1.5141	0.0348	1.5134	0.0312	1.5132	0.0329
			1.5104	0.0358	1.5116	0.0371	1.5106	0.0360	1.5105	0.0336	1.5104	0.0375
4	30	20	1.5324	0.1200	1.5359	0.1270	1.5339	0.1237	1.5341	0.1173	1.5343	0.1214
			1.5367	0.1297	1.5433	0.1281	1.5427	0.1268	1.5421	0.1296	1.5415	0.1246
		25	1.5327	0.0970	1.5354	0.0980	1.5340	0.0960	1.5338	1.0002	1.5343	0.0964
			1.5292	0.0983	1.5347	0.1031	1.5339	0.1059	1.5331	0.1020	1.5331	0.1025
	50	40	1.5196	0.0579	1.5235	0.0579	1.5218	0.0521	1.5221	0.0561	1.5220	0.0524
			1.5183	0.0633	1.5149	0.0652	1.5136	0.0565	1.5133	0.0628	1.5136	0.0586
		45	1.5168	0.0580	1.5182	0.0552	1.5168	0.0532	1.5170	0.0521	1.5174	0.0542
			1.5195	0.0584	1.5197	0.0598	1.5188	0.0570	1.5192	0.0579	1.5186	0.0559
	80	60	1.5109	0.0403	1.5097	0.0402	1.5091	0.0405	1.5086	0.0407	1.5085	0.0358
			1.5088	0.0414	1.5098	0.0430	1.5088	0.0435	1.5083	0.0412	1.5080	0.0582
		70	1.5121	0.0326	1.5160	0.0372	1.5150	0.0331	1.5151	0.0299	1.5156	0.0331
			1.5105	0.0334	1.5124	0.0353	1.5108	0.0362	1.5113	0.0361	1.5105	0.0371

**Table A3:** The results of Bayesian estimates with non-informative priors for λ using the Lindley method.

*T*	*n*	*m*	${\hat{\sigma }}_{\mathrm{SE}}$		${\hat{\lambda }}_{\mathrm{LL}}$				${\hat{\lambda }}_{\mathrm{GE}}$			
					$p=\frac{1}{2}$		p=1		$q=-\frac{1}{2}$		$q=\frac{1}{2}$	
			**VALUE**	**MSE**	**VALUE**	**MSE**	**VALUE**	**MSE**	**VALUE**	**MSE**	**VALUE**	**MSE**
2	30	20	1.1021	0.1065	1.1024	0.1075	1.1019	0.1062	1.1017	0.1059	1.1018	0.1062
			1.1064	0.1129	1.1071	0.1147	1.1063	0.1127	1.1071	0.1134	1.1055	0.1125
		25	1.1024	0.1012	1.1021	0.1003	1.1019	0.1003	1.1006	0.1000	1.1019	0.0991
			1.0999	0.1014	1.0996	0.1022	1.0990	0.1022	1.1005	0.1016	1.0995	0.1016
	50	40	1.0594	0.0524	1.0604	0.0539	1.0600	0.0523	1.0604	0.0514	1.0590	0.0524
			1.0563	0.0491	1.0561	0.0489	1.0558	0.0481	1.0547	0.0487	1.0561	0.0487
		45	1.0578	0.0474	1.0570	0.0461	1.0567	0.0463	1.0572	0.0479	1.0565	0.0462
			1.0543	0.0454	1.0552	0.0439	1.0551	0.0449	1.0534	0.0454	1.0551	0.0444
	80	60	1.0393	0.0282	1.0393	0.0285	1.0392	0.0313	1.0387	0.0282	1.0400	0.0290
			1.0328	0.0267	1.0325	0.0260	1.0330	0.0275	1.0325	0.0255	1.0339	0.0256
		70	1.0342	0.0266	1.0338	0.0264	1.0347	0.0261	1.0330	0.0259	1.0341	0.0269
			1.0327	0.0250	1.0324	0.0250	1.0335	0.0268	1.0336	0.0256	1.0317	0.0257
4	30	20	1.1121	0.1138	1.1123	0.1120	1.1127	0.1122	1.1124	0.1135	1.1119	0.1128
			1.1145	0.1176	1.1140	0.1158	1.1148	0.1164	1.1144	0.1157	1.1140	0.1155
		25	1.1053	0.1032	1.1057	0.1028	1.1060	0.1024	1.1055	0.1031	1.1054	0.1023
			1.1034	0.1059	1.1048	0.1038	1.1034	0.1054	1.1047	0.1042	1.1040	0.1049
	50	40	1.0566	0.0501	1.0560	0.0490	1.0558	0.0493	1.0568	0.0493	1.0560	0.0490
			1.0493	0.0450	1.0491	0.0448	1.0487	0.0446	1.0491	0.0460	1.0492	0.0449
		45	1.0509	0.0462	1.0517	0.0470	1.0517	0.0473	1.0501	0.0459	1.0507	0.0459
			1.0527	0.0448	1.0530	0.0449	1.0525	0.0450	1.0527	0.0457	1.0527	0.0436
	80	60	1.0338	0.0297	1.0337	0.0297	1.0340	0.0293	1.0330	0.0297	1.0340	0.0292
			1.0297	0.0290	1.0290	0.0272	1.0300	0.0278	1.0288	0.0287	1.0287	0.0285
		70	1.0326	0.0260	1.0331	0.0268	1.0334	0.0264	1.0336	0.0252	1.0340	0.0263
			1.0328	0.0263	1.0324	0.0253	1.0324	0.0258	1.0332	0.0262	1.0316	0.0247

**Table A4:** The results of Bayesian estimates with non-informative priors for σ using importance sampling.

*T*	*n*	*m*	${\hat{\lambda }}_{\mathrm{SE}}$		${\hat{\lambda }}_{\mathrm{LL}}$				${\hat{\lambda }}_{\mathrm{GE}}$			
					$p=\frac{1}{2}$		p=1		$q=-\frac{1}{2}$		$q=\frac{1}{2}$	
			**VALUE**	**MSE**	**VALUE**	**MSE**	**VALUE**	**MSE**	**VALUE**	**MSE**	**VALUE**	**MSE**
2	30	20	1.5302	0.1207	1.4709	0.1089	1.4641	0.1172	1.5326	0.1205	1.5251	0.1298
			1.5382	0.1419	1.5321	0.1323	1.5292	0.1391	1.5328	0.1356	1.5307	0.1438
		25	1.5305	0.0892	1.5301	0.0882	1.5246	0.0815	1.5327	0.0822	1.5299	0.0844
			1.5393	0.0997	1.5327	0.0989	1.5257	0.0907	1.5311	0.0925	1.5279	0.0942
	50	40	1.5293	0.0595	1.5275	0.0616	1.5244	0.0589	1.5203	0.0585	1.5242	0.0558
			1.5253	0.0631	1.5220	0.0602	1.5291	0.0675	1.5253	0.0593	1.5281	0.0653
		45	1.5269	0.0568	1.5296	0.0567	1.5266	0.0541	1.5290	0.0557	1.5237	0.0536
			1.5253	0.0562	1.5270	0.0527	1.5248	0.0548	1.5277	0.0571	1.5226	0.0550
	80	60	1.5126	0.0403	1.5125	0.0357	1.5109	0.0396	1.5179	0.0389	1.5147	0.0376
			1.5098	0.0428	1.5151	0.0404	1.5135	0.0422	1.5140	0.0444	1.5103	0.0431
		70	1.5117	0.0350	1.5091	0.0340	1.5078	0.0331	1.5130	0.0340	1.5104	0.0360
			1.5148	0.0371	1.5078	0.0310	1.5064	0.0331	1.5108	0.0348	1.5082	0.0328
4	30	20	1.5288	0.1211	1.5313	0.1121	1.5323	0.1185	1.5330	0.1145	1.5321	0.1225
			1.5378	0.1372	1.5324	0.1366	1.5302	0.1309	1.5363	0.1335	1.5356	0.1371
		25	1.5298	0.0943	1.5352	0.0906	1.5285	0.0924	1.5227	0.0973	1.5202	0.0990
			1.5311	0.1021	1.5241	0.1081	1.5370	0.0991	1.5333	0.1054	1.5206	0.1078
	50	40	1.5265	0.0515	1.5250	0.0556	1.5222	0.0553	1.5274	0.0590	1.5212	0.0562
			1.5250	0.0633	1.5241	0.0665	1.5221	0.0634	1.5253	0.0609	1.5290	0.0683
		45	1.5266	0.0579	1.5241	0.0507	1.5214	0.0584	1.5290	0.0558	1.5239	0.0537
			1.5240	0.0596	1.5262	0.0556	1.5239	0.0537	1.5263	0.0575	1.5212	0.0553
	80	60	1.5167	0.0444	1.5101	0.0480	1.5184	0.0468	1.5117	0.0430	1.5183	0.0416
			1.51198	0.0479	1.5145	0.0499	1.5130	0.0389	1.5162	0.0465	1.5148	0.0451
		70	1.5083	0.0337	1.5108	0.0355	1.5095	0.0346	1.5144	0.0327	1.5118	0.0318
			1.5082	0.0353	1.5069	0.0334	1.5157	0.0356	1.5146	0.0342	1.5121	0.0332

**Table A5:** The results of Bayesian estimates with non-informative priors for λ using importance sampling.

*T*	*n*	*m*	${\hat{\lambda }}_{\mathrm{SE}}$		${\hat{\lambda }}_{\mathrm{LL}}$				${\hat{\lambda }}_{\mathrm{GE}}$			
					$p=\frac{1}{2}$		p=1		$q=-\frac{1}{2}$		$q=\frac{1}{2}$	
			**VALUE**	**MSE**	**VALUE**	**MSE**	**VALUE**	**MSE**	**VALUE**	**MSE**	**VALUE**	**MSE**
2	30	20	1.1076	0.1033	1.1079	0.1053	1.1042	0.1003	1.1002	0.1083	1.1004	0.1028
			1.1061	0.1110	1.1086	0.1157	1.1006	0.1173	1.1004	0.1183	1.1078	0.1193
		25	1.1063	0.0962	1.1023	0.0927	1.1013	0.0914	1.1031	0.0941	0.9085	0.0932
			1.1089	0.0966	1.1040	0.0918	1.1026	0.0934	1.1051	0.0939	1.1006	0.0948
	50	40	1.0589	0.0526	1.0513	0.0498	0.9547	0.0501	1.0514	0.0503	0.9470	0.0508
			1.0532	0.0468	1.0561	0.0459	0.9493	0.0478	1.0561	0.0463	0.9517	0.0484
		45	0.9475	0.0432	0.9508	0.0429	0.9531	0.0424	0.9507	0.0432	0.9561	0.0433
			1.0507	0.0459	1.0518	0.0453	0.9554	0.0457	1.0519	0.0456	0.9585	0.0463
	80	60	0.9648	0.0282	0.9702	0.0279	0.9723	0.0271	0.9702	0.0281	0.9774	0.0276
			0.9694	0.0265	0.9755	0.0264	0.9793	0.0253	0.9754	0.0265	0.9749	0.0257
		70	0.9773	0.0245	0.9737	0.0245	0.9774	0.0257	0.9735	0.0246	0.9731	0.0262
			0.9731	0.0261	0.9797	0.0261	0.9705	0.0261	0.9796	0.0262	0.9764	0.0265
4	30	20	1.1117	0.1115	1.1143	0.1158	1.1164	0.1108	1.1157	0.1182	1.1126	0.1130
			1.1187	0.1111	1.1155	0.1153	1.1134	0.1155	1.1168	0.1175	1.1112	0.1172
		25	1.1080	0.1096	1.1032	0.1047	1.1046	0.1084	1.1045	0.1066	1.1021	0.1099
			1.1042	0.1003	1.1078	0.1096	1.1024	0.1081	1.1087	0.1013	0.9001	0.1097
	50	40	1.0540	0.0498	0.9489	0.0491	1.0527	0.0522	0.9489	0.0495	0.9450	0.0533
			1.0576	0.0488	1.0502	0.0479	1.0552	0.0396	1.0504	0.0384	0.9479	0.0430
		45	1.0537	0.0482	0.9468	0.0475	0.9548	0.0449	0.9468	0.0479	0.9577	0.0456
			1.0544	0.0454	0.9477	0.0449	0.9587	0.0447	0.9477	0.0452	0.9518	0.0452
	80	60	0.9650	0.0307	0.9607	0.0306	0.9682	0.0292	0.9706	0.0307	0.9734	0.0366
			0.9675	0.0268	0.9636	0.0267	0.9647	0.0259	0.9635	0.0268	0.9703	0.0363
		70	0.9630	0.0245	0.9693	0.0245	0.9684	0.0232	0.9692	0.0246	0.9642	0.0337
			0.9639	0.0261	0.9603	0.0261	0.9622	0.0234	0.9702	0.0262	0.9683	0.0338

## Appendix C. The Simulation Results of Bayesian Estimates with Informative Priors

**Table A6:** The results of Bayesian estimates with informative priors for σ using the Lindley method.

*T*	*n*	*m*	${\hat{\sigma }}_{\mathrm{SE}}$		${\hat{\sigma }}_{\mathrm{LL}}$				${\hat{\sigma }}_{\mathrm{GE}}$			
					$p=\frac{1}{2}$		p=1		$q=-\frac{1}{2}$		$q=\frac{1}{2}$	
			**VALUE**	**MSE**	**VALUE**	**MSE**	**VALUE**	**MSE**	**VALUE**	**MSE**	**VALUE**	**MSE**
2	30	20	1.5319	0.1153	1.5307	0.1125	1.5315	0.1259	1.4699	0.1010	1.5329	0.1187
			1.5352	0.1254	1.5338	0.1176	1.5310	0.1301	1.4637	0.1042	1.5305	0.1271
		25	1.5235	0.0895	1.5298	0.0890	1.5212	0.1117	1.4737	0.0750	1.5295	0.0937
			1.5295	0.1102	1.5257	0.0984	1.5205	0.1218	1.4741	0.0821	1.5377	0.1018
	50	40	1.5203	0.0542	1.5217	0.0499	1.5245	0.0516	1.4785	0.0531	1.5235	0.0567
			1.5281	0.0566	1.5265	0.0519	1.5248	0.0593	1.4780	0.0545	1.5230	0.0632
		45	1.5156	0.0417	1.5166	0.0485	1.5105	0.0439	1.4841	0.0440	1.5106	0.0498
			1.5250	0.0473	1.5256	0.0535	1.5234	0.0448	1.4834	0.0476	1.5236	0.0509
	80	60	1.5059	0.0357	1.5114	0.0339	1.5133	0.0346	1.4961	0.0315	1.5165	0.0325
			1.5154	0.0399	1.5151	0.0436	1.5149	0.0409	1.4992	0.0316	1.5176	0.0462
		70	1.5033	0.0289	1.5010	0.0276	1.5052	0.0297	1.4993	0.0255	1.5095	0.0283
			1.5059	0.0294	1.5034	0.0280	1.5023	0.0307	1.4934	0.0257	1.5164	0.0291
4	30	20	1.5324	0.1234	1.5312	0.1144	1.5381	0.1292	1.4606	0.1025	1.5305	0.1202
			1.5371	0.1235	1.5356	0.1154	1.5306	0.1364	1.4654	0.1026	1.5391	0.1250
		25	1.5265	0.1056	1.5227	0.0945	1.5261	0.1094	1.4779	0.0775	1.5244	0.0920
			1.5242	0.1064	1.5305	0.0951	1.5227	0.1115	1.4753	0.0789	1.5311	0.1141
	50	40	1.5173	0.0624	1.5258	0.0579	1.5239	0.0630	1.4772	0.0511	1.5227	0.0577
			1.5198	0.0640	1.5284	0.0594	1.5290	0.0679	1.4750	0.0519	1.5275	0.0622
		45	1.5091	0.0551	1.5199	0.0417	1.5129	0.0544	1.4853	0.0467	1.5132	0.0505
			1.5121	0.0530	1.5130	0.0495	1.5146	0.0507	1.4899	0.0439	1.5253	0.0473
	80	60	1.5107	0.0380	1.5162	0.0360	1.5133	0.0346	1.4911	0.0320	1.5125	0.0325
			1.5148	0.0471	1.5140	0.0404	1.5108	0.0406	1.4907	0.0329	1.5134	0.0457
		70	1.5061	0.0284	1.5037	0.0270	1.5059	0.0278	1.4954	0.0260	1.5101	0.0264
			1.5078	0.0300	1.5054	0.0285	1.5059	0.0318	1.4925	0.0248	1.5100	0.0302

**Table A7:** The results of Bayesian estimates with informative priors for λ using the Lindley method.

*T*	*n*	*m*	${\hat{\lambda }}_{\mathrm{SE}}$		${\hat{\lambda }}_{\mathrm{LL}}$				${\hat{\lambda }}_{\mathrm{GE}}$			
					$p=\frac{1}{2}$		p=1		$q=-\frac{1}{2}$		$q=\frac{1}{2}$	
			**VALUE**	**MSE**	**VALUE**	**MSE**	**VALUE**	**MSE**	**VALUE**	**MSE**	**VALUE**	**MSE**
2	30	20	1.0909	0.1012	1.0895	0.0995	1.0908	0.0923	1.0887	0.0983	1.0915	0.0934
			1.0961	0.0975	1.0883	0.0976	1.0856	0.0922	1.0873	0.0962	1.0795	0.0920
		25	1.0878	0.0969	1.0772	0.0957	0.9273	0.0932	1.0766	0.0949	1.0773	0.0924
			1.0765	0.0908	1.0697	0.0929	0.9278	0.0919	1.0689	0.0932	1.6983	0.0910
	50	40	1.0664	0.0498	1.0496	0.0491	0.9424	0.0437	1.0594	0.0487	0.9490	0.0433
			1.0579	0.0435	1.0411	0.0431	0.9548	0.0402	1.0510	0.0428	0.9518	0.0397
		45	0.9952	0.0385	0.9691	0.0385	0.9548	0.0337	0.9511	0.0383	0.9533	0.0333
			1.0547	0.0373	1.0362	0.0368	0.9679	0.0313	1.0480	0.0366	0.9545	0.0310
	80	60	1.0476	0.0191	1.0328	0.0190	0.9737	0.0134	1.0328	0.0188	0.9684	0.0132
			1.0302	0.0141	0.9763	0.0143	1.0312	0.0135	0.9765	0.0142	1.0254	0.0144
		70	0.9775	0.0135	0.9737	0.0134	0.9809	0.0137	0.9738	0.0133	0.9849	0.0133
			0.9752	0.0119	0.9763	0.0119	0.9852	0.0139	0.9814	0.0119	0.9790	0.0115
4	30	20	1.0927	0.1037	1.0892	0.1017	0.9186	0.0996	1.0882	0.1006	1.0897	0.0967
			1.0899	0.0965	1.0882	0.0957	1.0833	0.0977	1.0873	0.0937	1.0835	0.0967
		25	0.9271	0.0930	0.9265	0.0925	0.9202	0.0861	0.9262	0.0918	1.0708	0.0955
			1.0835	0.0858	1.0629	0.0850	1.0760	0.0838	1.0726	0.0844	1.0653	0.0826
	50	40	1.0565	0.0408	1.0683	0.0401	1.0591	0.0317	1.0520	0.0397	1.0559	0.0317
			1.0506	0.0302	0.9340	0.0300	0.9599	0.0291	0.9539	0.0298	1.0465	0.0289
		45	1.0546	0.0323	1.0482	0.0320	0.9576	0.0314	1.0481	0.0317	0.9542	0.0310
			1.0495	0.0286	1.0433	0.0283	1.0449	0.0254	1.0432	0.0280	1.0412	0.0253
	80	60	0.9729	0.0152	0.9685	0.0152	0.9652	0.0135	0.9786	0.0151	0.9797	0.0142
			1.0327	0.0151	0.9688	0.0150	0.9670	0.0184	0.9787	0.0149	1.0210	0.0132
		70	0.9765	0.0120	0.9725	0.0129	0.9708	0.0125	0.9825	0.0129	0.9847	0.0120
			1.0225	0.0120	0.9789	0.0125	0.9740	0.0120	0.9889	0.0128	0.9780	0.0119

**Table A8:** The results of Bayesian estimates with informative priors for σ using importance sampling.

*T*	*n*	*m*	${\hat{\sigma }}_{\mathrm{SE}}$		${\hat{\sigma }}_{\mathrm{LL}}$				${\hat{\sigma }}_{\mathrm{GE}}$			
					$p=\frac{1}{2}$		p=1		$q=-\frac{1}{2}$		$q=\frac{1}{2}$	
			**VALUE**	**MSE**	**VALUE**	**MSE**	**VALUE**	**MSE**	**VALUE**	**MSE**	**VALUE**	**MSE**
2	30	20	1.5380	0.0790	1.5336	0.0678	1.5337	0.0745	1.5379	0.0694	1.5336	0.0632
			1.5347	0.0985	1.5394	0.0848	1.5353	0.0918	1.5320	0.0845	1.5342	0.0778
		25	1.5352	0.0729	1.5304	0.0675	1.5313	0.0691	1.5282	0.0652	1.5269	0.0561
			1.5303	0.0796	1.5362	0.0635	1.5361	0.0757	1.5368	0.0715	1.5330	0.0598
	50	40	1.5296	0.0522	1.5210	0.0471	1.5222	0.0505	1.5274	0.0487	1.5295	0.0457
			1.5332	0.0476	1.5289	0.0439	1.5260	0.0461	1.5215	0.0445	1.5271	0.0424
		45	1.5207	0.0439	1.5276	0.0377	1.5216	0.0427	1.5177	0.0413	1.5158	0.0362
			1.5270	0.0463	1.5201	0.0437	1.5279	0.0443	1.5221	0.0425	1.5286	0.0421
	80	60	1.5188	0.0271	1.5151	0.0261	1.5139	0.0261	1.5109	0.0252	1.5108	0.0253
			1.5201	0.0314	1.5114	0.0264	1.5142	0.0301	1.5044	0.0290	1.5113	0.0255
		70	1.5075	0.0214	1.5038	0.0187	1.5037	0.0205	1.5022	0.0198	1.5088	0.0181
			1.5117	0.0217	1.5071	0.0196	1.5068	0.0210	1.5044	0.0204	1.5110	0.0190
4	30	20	1.5322	0.0527	1.5371	0.0737	1.5351	0.0494	1.5345	0.0461	1.5333	0.0685
			1.5331	0.0828	1.5309	0.0931	1.5346	0.0777	1.5325	0.0718	1.5344	0.0853
		25	1.5277	0.0763	1.5292	0.0849	1.5234	0.0732	1.5243	0.0665	1.5249	0.0596
			1.5261	0.0771	1.5321	0.0639	1.5334	0.0817	1.5344	0.0684	1.5358	0.0698
	50	40	1.5254	0.0453	1.5203	0.0392	1.5263	0.0434	1.5206	0.0416	1.5191	0.0378
			1.5249	0.0453	1.5207	0.0465	1.5268	0.0440	1.5219	0.0424	1.5195	0.0451
		45	1.5233	0.0352	1.5204	0.0346	1.5261	0.0345	1.5219	0.0336	1.5228	0.0394
			1.5157	0.0405	1.5133	0.0354	1.5183	0.0391	1.5136	0.0377	1.5156	0.0342
	80	60	1.5168	0.0270	1.5126	0.0217	1.5116	0.0258	1.5093	0.0247	1.5114	0.0210
			1.5127	0.0278	1.5161	0.0229	1.5130	0.0268	1.5105	0.0248	1.5148	0.0221
		70	1.5072	0.0227	1.5108	0.0220	1.5132	0.0220	1.5108	0.0214	1.5097	0.0214
			1.5024	0.0254	1.5088	0.0261	1.5082	0.0244	1.5055	0.0236	1.5078	0.0235

**Table A9:** The results of Bayesian estimates with informative priors for λ using importance sampling.

*T*	*n*	*m*	${\hat{\lambda }}_{\mathrm{SE}}$		${\hat{\lambda }}_{\mathrm{LL}}$				${\hat{\lambda }}_{\mathrm{GE}}$			
					$p=\frac{1}{2}$		p=1		$q=-\frac{1}{2}$		$q=\frac{1}{2}$	
			**VALUE**	**MSE**	**VALUE**	**MSE**	**VALUE**	**MSE**	**VALUE**	**MSE**	**VALUE**	**MSE**
2	30	20	1.0909	0.1012	1.0895	0.0995	1.0908	0.0923	1.0887	0.0983	1.0915	0.0934
			1.0961	0.0975	1.0883	0.0976	1.0856	0.0922	1.0873	0.0962	1.0795	0.0920
		25	1.0878	0.0969	1.0772	0.0957	0.9273	0.0932	1.0766	0.0949	1.0773	0.0924
			1.0765	0.0908	1.0697	0.0929	0.9278	0.0919	1.0689	0.0932	1.6983	0.0910
	50	40	1.0664	0.0498	1.0496	0.0491	0.9424	0.0437	1.0594	0.0487	0.9490	0.0433
			1.0579	0.0435	1.0411	0.0431	0.9548	0.0402	1.0510	0.0428	0.9518	0.0397
		45	0.9952	0.0385	0.9691	0.0385	0.9548	0.0337	0.9511	0.0383	0.9533	0.0333
			1.0547	0.0373	1.0362	0.0368	0.9679	0.0313	1.0480	0.0366	0.9545	0.0310
	80	60	1.0476	0.0191	1.0328	0.0190	0.9737	0.0134	1.0328	0.0188	0.9684	0.0132
			1.0302	0.0141	0.9763	0.0143	1.0312	0.0135	0.9765	0.0142	1.0254	0.0144
		70	0.9775	0.0135	0.9737	0.0134	0.9809	0.0137	0.9738	0.0133	0.9849	0.0133
			0.9752	0.0119	0.9763	0.0119	0.9852	0.0139	0.9814	0.0119	0.9790	0.0115
4	30	20	1.0927	0.1037	1.0892	0.1017	0.9186	0.0996	1.0882	0.1006	1.0897	0.0967
			1.0899	0.0965	1.0882	0.0957	1.0833	0.0977	1.0873	0.0937	1.0835	0.0967
		25	0.9271	0.0930	0.9265	0.0925	0.9202	0.0861	0.9262	0.0918	1.0708	0.0955
			1.0835	0.0858	1.0629	0.0850	1.0760	0.0838	1.0726	0.0844	1.0653	0.0826
	50	40	1.0565	0.0408	1.0683	0.0401	1.0591	0.0317	1.0520	0.0397	1.0559	0.0317
			1.0506	0.0302	0.9340	0.0300	0.9599	0.0291	0.9539	0.0298	1.0465	0.0289
		45	1.0546	0.0323	1.0482	0.0320	0.9576	0.0314	1.0481	0.0317	0.9542	0.0310
			1.0495	0.0286	1.0433	0.0283	1.0449	0.0254	1.0432	0.0280	1.0412	0.0253
	80	60	0.9729	0.0152	0.9685	0.0152	0.9652	0.0135	0.9786	0.0151	0.9797	0.0142
			1.0327	0.0151	0.9688	0.0150	0.9670	0.0184	0.9787	0.0149	1.0210	0.0132
		70	0.9765	0.0120	0.9725	0.0129	0.9708	0.0125	0.9825	0.0129	0.9847	0.0120
			1.0225	0.0120	0.9789	0.0125	0.9740	0.0120	0.9889	0.0128	0.9780	0.0119

## Appendix D. The Simulation Results of All Intervals

**Table A10:** The simulation results of five intervals for σ.

*T*	*n*	*m*	Sch	ACI		boop-p		boot-t		HPD			
										non-infor		infor	
				ML	CR	ML	CR	ML	CR	ML	CR	ML	CR
2	30	20	I	1.3317	0.8903	1.2490	0.8847	1.2206	0.9163	1.3226	0.8883	1.1232	0.8277
			II	1.3911	0.8893	1.4017	0.8917	1.3817	0.9047	1.3757	0.8891	1.1982	0.8383
		25	I	1.1989	0.8923	1.2019	0.8970	1.1141	0.9210	1.1695	0.8887	1.0093	0.8410
			II	1.2153	0.9083	1.2504	0.8970	1.1093	0.9157	1.1835	0.9038	1.0141	0.8477
	50	40	I	0.9626	0.9160	0.9915	0.9056	0.8741	0.9440	0.9483	0.9106	0.7634	0.8580
			II	0.9712	0.9280	0.9817	0.9193	0.9596	0.9253	0.9382	0.9214	0.7762	0.8767
		45	I	0.9131	0.9260	0.9540	0.9220	0.8097	0.9440	0.9080	0.9219	0.7233	0.8773
			II	0.9126	0.9250	0.9416	0.9147	0.8124	0.9433	0.8982	0.9237	0.7168	0.8753
	80	60	I	0.7919	0.9293	0.8117	0.9180	0.6917	0.9527	0.7732	0.9230	0.5902	0.8700
			II	0.8053	0.9283	0.7979	0.9293	0.7052	0.9520	0.7863	0.9272	0.6035	0.8800
		70	I	0.7347	0.9317	0.7573	0.9396	0.6328	0.9500	0.7059	0.9252	0.5310	0.8847
			II	0.7313	0.9247	0.7766	0.9380	0.6373	0.9487	0.6982	0.9228	0.5385	0.8773
4	30	20	I	1.3309	0.8943	1.3719	0.8897	1.2348	0.9007	1.3268	0.8903	1.1348	0.8367
			II	1.3853	0.8810	1.3972	0.8967	1.3912	0.9150	1.3740	0.8800	1.1838	0.8273
		25	I	1.1981	0.9020	1.2543	0.8980	1.1131	0.9257	1.1897	0.8983	1.0104	0.8243
			II	1.2229	0.9050	1.2726	0.9113	1.1227	0.9190	1.1945	0.9015	1.0109	0.8387
	50	40	I	0.9621	0.9200	0.9906	0.9115	0.8622	0.9510	0.9562	0.9149	0.7614	0.8453
			II	0.9795	0.9230	0.9850	0.9160	0.8725	0.9487	0.9656	0.9202	0.7693	0.8513
		45	I	0.9129	0.9223	0.9343	0.9267	0.8169	0.9467	0.9111	0.9183	0.7151	0.8680
			II	0.9114	0.9217	0.9162	0.9273	0.8148	0.9500	0.8882	0.9183	0.7154	0.8760
	80	60	I	0.7892	0.9340	0.8126	0.9438	0.6891	0.9467	0.7601	0.9314	0.5927	0.8673
			II	0.8000	0.9210	0.8165	0.9247	0.7062	0.9560	0.7723	0.9154	0.6034	0.8647
		70	I	0.7354	0.9300	0.7443	0.9173	0.6323	0.9580	0.7286	0.9281	0.5374	0.8727
			II	0.7336	0.9310	0.7372	0.9333	0.6357	0.9520	0.7175	0.9258	0.5355	0.8713

**Table A11:** The simulation results of five intervals for λ.

*T*	*n*	*m*	Sch	ACI		boop-p		boot-t		HPD			
										non-infor		infor	
				ML	CR	ML	CR	ML	CR	ML	CR	ML	CR
2	30	20	I	1.1725	0.9757	1.2475	0.9683	1.1384	0.9727	1.1518	0.9740	0.9855	0.9333
			II	1.1256	0.9760	1.1127	0.9720	1.0805	0.9767	1.1010	0.9757	0.9149	0.9343
		25	I	1.0975	0.9670	1.1181	0.9683	1.0467	0.9700	1.0800	0.9662	0.8888	0.9310
			II	1.0547	0.9737	1.0705	0.9660	1.0307	0.9737	1.0333	0.9691	0.8649	0.9290
	50	40	I	0.8137	0.9563	0.9125	0.9570	0.7454	0.9665	0.8130	0.9531	0.8149	0.9165
			II	0.7850	0.9600	0.7832	0.9593	0.7540	0.9697	0.7843	0.9599	0.6828	0.9167
		45	I	0.7861	0.9610	0.7972	0.9620	0.7409	0.9687	0.7572	0.9583	0.5714	0.9127
			II	0.7715	0.9540	0.7716	0.9533	0.7373	0.9597	0.7483	0.9474	0.5620	0.9207
	80	60	I	0.6444	0.9553	0.6664	0.9560	0.6028	0.9680	0.6177	0.9524	0.4456	0.9180
			II	0.6072	0.9543	0.6552	0.9467	0.5687	0.9510	0.5985	0.9509	0.4104	0.9213
		70	I	0.6071	0.9533	0.6263	0.9593	0.5707	0.9503	0.5891	0.9521	0.4114	0.9193
			II	0.6119	0.9547	0.6244	0.9613	0.5569	0.9507	0.5953	0.9486	0.3963	0.9120
4	30	20	I	1.1804	0.9730	1.2876	0.9690	1.0701	0.9767	1.1675	0.9673	0.9649	0.9340
			II	1.1162	0.9670	1.1590	0.9730	1.0710	0.9773	1.1049	0.9643	0.9150	0.9300
		25	I	1.0862	0.9660	1.1597	0.9707	1.0303	0.9750	1.0706	0.9621	0.8776	0.9260
			II	1.0459	0.9667	1.0828	0.9703	1.0023	0.9793	1.0172	0.9643	0.8611	0.9293
	50	40	I	0.8169	0.9573	0.9245	0.9595	0.7183	0.9745	0.7907	0.9543	0.6126	0.9153
			II	0.7839	0.9553	0.7913	0.9620	0.7482	0.9727	0.7708	0.9530	0.5899	0.9193
		45	I	0.7806	0.9543	0.7859	0.9627	0.7277	0.9613	0.7739	0.9525	0.5706	0.9113
			II	0.7724	0.9610	0.7674	0.9513	0.7157	0.9760	0.7442	0.9548	0.5634	0.9180
	80	60	I	0.6437	0.9593	0.6638	0.9520	0.5828	0.9647	0.6175	0.9584	0.4463	0.9167
			II	0.6125	0.9473	0.6484	0.9547	0.5991	0.9600	0.5976	0.9432	0.4108	0.9107
		70	I	0.6094	0.9550	0.6255	0.9587	0.5227	0.9687	0.5813	0.9493	0.4106	0.9067
			II	0.6124	0.9523	0.5959	0.9600	0.5347	0.9667	0.6093	0.9471	0.3967	0.9187
